# FABP3 Deficiency Exacerbates Metabolic Derangement in Cardiac Hypertrophy and Heart Failure via PPARα Pathway

**DOI:** 10.3389/fcvm.2021.722908

**Published:** 2021-08-12

**Authors:** Lingfang Zhuang, Ye Mao, Zizhu Liu, Chenni Li, Qi Jin, Lin Lu, Rong Tao, Xiaoxiang Yan, Kang Chen

**Affiliations:** ^1^Department of Vascular and Cardiology, Ruijin Hospital, Shanghai Jiao Tong University School of Medicine, Shanghai, China; ^2^Institute of Cardiovascular Diseases, Shanghai Jiao Tong University School of Medicine, Shanghai, China; ^3^Department of Health Management Center, Ruijin Hospital Lu Wan Branch, Shanghai Jiao Tong University School of Medicine, Shanghai, China

**Keywords:** cardiac hypertrophy, PPARα, HFABP, metabolism, FAO, glycolysis

## Abstract

**Background:** Cardiac hypertrophy was accompanied by various cardiovascular diseases (CVDs), and due to the high global incidence and mortality of CVDs, it has become increasingly critical to characterize the pathogenesis of cardiac hypertrophy. We aimed to determine the metabolic roles of fatty acid binding protein 3 (FABP3) on transverse aortic constriction (TAC)-induced cardiac hypertrophy.

**Methods and Results:** Transverse aortic constriction or Ang II treatment markedly upregulated Fabp3 expression. Notably, Fabp3 ablation aggravated TAC-induced cardiac hypertrophy and cardiac dysfunction. Multi-omics analysis revealed that Fabp3-deficient hearts exhibited disrupted metabolic signatures characterized by increased glycolysis, toxic lipid accumulation, and compromised fatty acid oxidation and ATP production under hypertrophic stimuli. Mechanistically, FABP3 mediated metabolic reprogramming by directly interacting with PPARα, which prevented its degradation and synergistically modulated its transcriptional activity on Mlycd and Gck. Finally, treatment with the PPARα agonist, fenofibrate, rescued the pro-hypertrophic effects of Fabp3 deficiency.

**Conclusions:** Collectively, these findings reveal the indispensable roles of the FABP3–PPARα axis on metabolic homeostasis and the development of hypertrophy, which sheds new light on the treatment of hypertrophy.

## Introduction

Cardiovascular diseases (CVDs) have become the primary cause of adult death worldwide (1). Of note, cardiac hypertrophy is induced by various CVDs, such as hypertension, hypertrophic cardiomyopathy (HCM), and storage diseases (associated with abnormal accumulation of lipid, glycogen, and misfolded proteins), and eventually lead to heart failure and death (2). Therefore, it becomes urgent to further elucidate the mechanism underlying the development of cardiac hypertrophy. To date, studies have confirmed that numerous mechanisms contribute to the onset and progression of cardiac hypertrophy, such as increased cell death and fibrosis, impaired protein and mitochondrial quality control, and reprogrammed metabolism (2, 3). Of note, the role of metabolic rewiring in hypertrophic progression has recently become a topic of research interest.

Through the use of advanced genomic technology, single-cell RNA sequencing (scRNA-seq), significant transcriptional differences in cellular metabolism have been described as one of the most profound aspects contributing to cardiac dysfunction (4). Specifically, an energy preference that shifted from fatty acid β-oxidation (FAO) to glucose metabolism, with the downregulation of FAO genes and subsequently the upregulation of glucose oxidation genes, has been described in pathophysiological conditions, such as hypertrophy and heart failure (5–7). Moreover, an increase in glucose consumption reportedly induced cardiac hypertrophy, while the preservation of FAO improved myocardial energetics and cardiac function (8, 9).

Fatty acid β-oxidation accounts for nearly 70% of ATP production in the postnatal heart, which underscores the pivotal role of fatty acid metabolism in maintaining heart function (10, 11). Unlike glucose, lipid species are insoluble and generally bind to lipid chaperones for transportation and utilization. Among them, fatty acid binding protein 3 (FABP3) is a small protein that is abundantly expressed in heart tissues and participates in cell metabolism by binding free long-chain fatty acids (LCFAs) and transporting them for cell metabolism, thereby protecting against lipid toxicity (12, 13). Additionally, FABP3 has been described in the context of cardiac hypertrophy, with a positive relationship being described between cellular and circulating levels of FABP3 and cardiac hypertrophy in patients and mice (14). However, the mechanism through which FABP3 affects cellular metabolic homeostasis and advances of cardiac hypertrophy remains poorly understood.

Accordingly, the present study aimed to determine the metabolic effects of FABP3 on transverse aortic constriction (TAC)-induced cardiac hypertrophy and heart failure using genetic mutant *Fabp3-*null mice. Our findings indicate that *Fabp3*-defect exacerbates cardiac hypertrophy and heart dysfunction, resulting in defective FAO, and increased glycolysis by impairing the PPARα signaling pathway. Furthermore, the agonist of PPARα, fenofibrate, attenuated TAC-induced cardiac hypertrophy in both wild-type (WT) and *Fabp3-*null mice. Collectively, this study for the first time demonstrates the indispensable role of FABP3 on metabolic homeostasis and the advance of hypertrophy and heart failure.

## Methods and Materials

### Animals and Generation of FABP3-KO Mice

C57BL/6 male mice were purchased from SLAC Laboratory Animal Co., Ltd. (Shanghai, China). Global *Fabp3* knockout (F3-KO) mice were generated using the CRISPR-Cas9 method as described previously (15) and housed in cages at room temperature and a 12-h light/dark cycle. All animal experimental procedures were approved by the Animal Care Committee of Shanghai Jiao Tong University School of Medicine. All animal procedures performed also conform to the guidelines from Directive 2010/63/EU of the European Parliament on the protection of animals used for scientific purposes or the current NIH guidelines. Expanded materials and methods are available in Supplementary Materials.

### Experimental Animals

The TAC model was conducted to induce pathological cardiac hypertrophy *in vivo*. Briefly, 8-week-old mice were anesthetized with isoflurane, intubated, and mechanically ventilated with a low concentration of isoflurane gas (1.0%), and the aortic arch was visualized and ligated with 6–0 silk suture against a 27-gauge needle, then the needle was removed and the chest was closed with 5–0 silk suture. For echocardiographic analysis, the mice were firstly anesthetized by inhalation of isoflurane (3%) at the beginning and continued without anesthetic to obtain a better heart rate. At the end of the experiments, the mice were sacrificed by intraperitoneally injecting a lethal dose of pentobarbital sodium (100 mg/kg) or were killed by cervical dislocation to obtain organs for further analysis.

### Statistical Analysis

Data are presented as the mean ± *SEM* or mean ± *SD*, which were performed using the GraphPad Prism software (version 7.0a, San Diego, CA, USA) or Rstudio. The statistical differences between two groups were analyzed with two-sided Student's *t*-test. For experiments with more than two groups, after confirming normality and homogeneity of variance, one-way analysis of variance followed by Tukey's *post-hoc* test was used for comparison in the situation of equal variance assumption; otherwise, the Games–Howell *post-hoc* test was used. Additionally, the Dunnett *post-hoc* test was applied to compare a single control group with the others. All statistical analyses were performed using the SPSS software (version 23; SPSS Inc., Chicago, USA). To confirm the survival rate between WT and F3-KO mice, Kaplan–Meier survival curve and log-rank statistics tests were performed in Rstudio using the Survminer package. For all statistical analysis, a *p*-value of < 0.05 was considered significant.

## Results

### Hypertrophic Stimuli Upregulate FABP3 Expression

**Figure 1:**
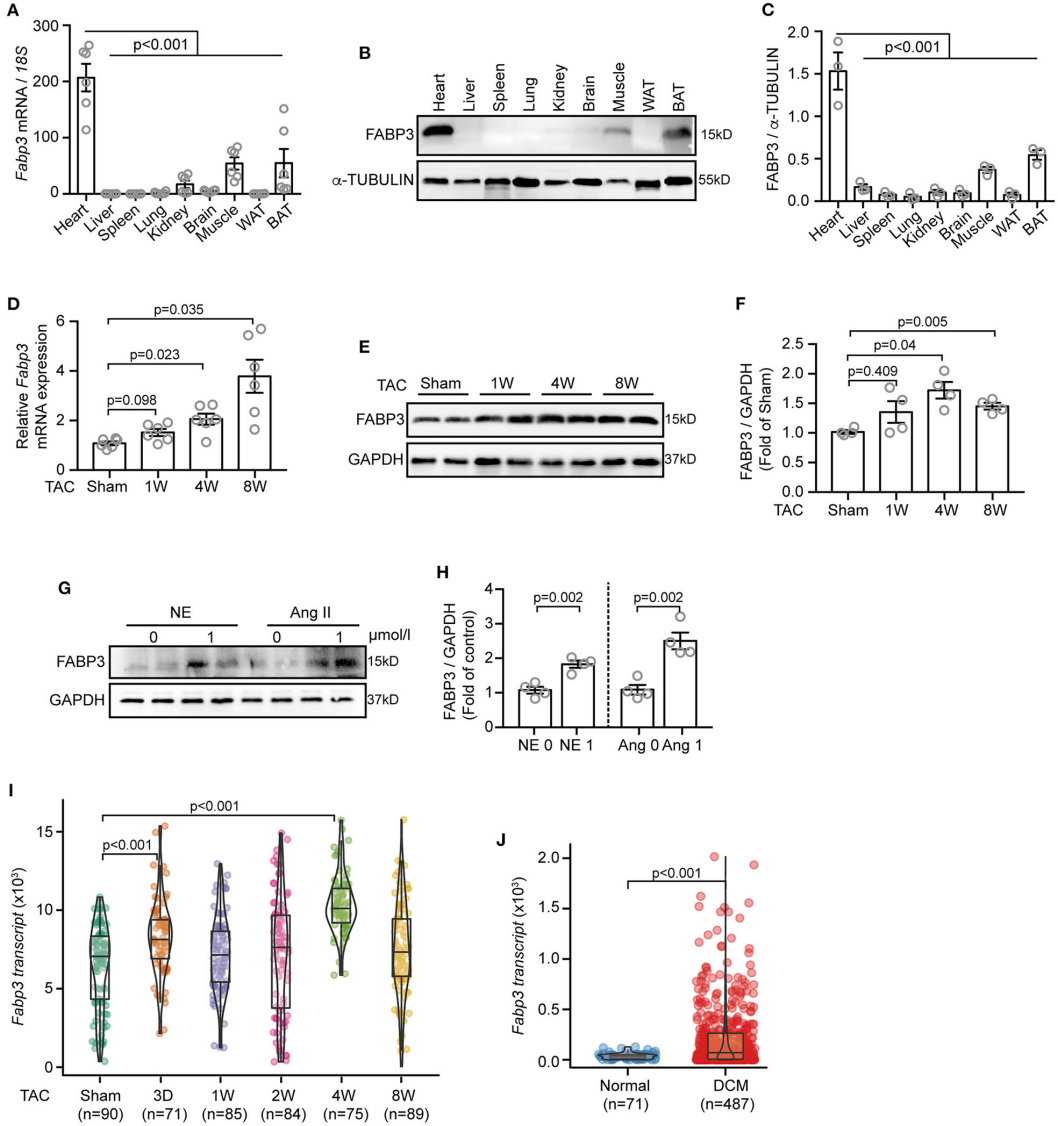
TAC or Ang II upregulates the FABP3 expression *in vivo* and *in vitro*. **(A–C)** Mouse tissues were extracted for qPCR and western blot analyses to determine *Fabp3* levels. **(A)** qPCR analysis of *Fabp3* mRNA in the indicated organs; *18s* was used as the control. **(B)** The FABP3 protein level in the corresponding tissue was determined using western blotting. **(C)** Quantification of FABP3 expression in **(B)**. (**D)** The mRNA level of *Fabp3* after TAC operation, as determined using qPCR. **(E)** Representative western blot images showing the FABP3 expression. **(F)** Quantification results for **(E)**. **(G)** NRVMs treated with NE or Ang II were subjected to western blotting assay to determine the FABP3 protein expression. **(H)** Quantification results of **(G)**. **(I,J)** Single cell RNA sequencing of TAC-operated murine hearts or heart samples from DCM and normal patients were recalculated for the transcriptional expression of *Fabp3* (GEO accession code: *GSE95143*). [**A**, *n* = 6; **C**, *n* = 3; **(A,C)** Dunnett's *post-hoc* test; **D**, *n* = 6; **F**, *n* = 4; **(D,F)** Games–Howell *post-hoc* test; **H**, *n* = 4; **(H,J)** Student's *t*-test; **(I)** Dunnett's *post-hoc* test].

Firstly, to gain an overall profile of FABP3 expression, we examined its mRNA and protein level in mouse tissues using quantitative polymerase chain reaction (qPCR) and western blotting assays. Our results showed that the FABP3 protein and *Fabp3* mRNA level was exclusively expressed in the hearts, BAT, and muscles, while its expression was rare in other organs (Figures 1A–C). Intriguingly, the hearts and BAT have been recognized for their distinct preference on fatty acids as energy sources (16), which underscores the important role of FABP3 in cardiac fatty acid metabolism. Then, by using TAC operation to induce cardiac hypertrophy *in vivo* and neurohormonal stimuli (NE or Ang II) *in vitro*, we observed increased FABP3 expression after TAC operations (Figures 1D–F) and after neurohormonal stimuli (Figures 1G,H).

Next, we assessed the transcriptional expression of *Fabp3* in the single-cell RNA-sequencing (scRNA-seq) datasets, which analyzed the transcriptional profile of murine cardiomyocytes after TAC surgery, and the scRNA-seq from dilated cardiomyopathy (DCM) or normal patients (17). Intriguingly, the scRNA-seq data in line with our *in vivo* and *in vitro* results showed significantly increased *Fabp3* expression at 4W after TAC surgery compared to the sham mice (Figure 1I) and more than five-fold higher *Fabp3* expression in DCM patients than their normal counterparts (Figure 1J). Taken together, these data suggest that FABP3 is expressed in heart tissues, which use fatty acids as a primary fuel substrate, and is upregulated *in vivo* and *in vitro* under hypertrophic stimuli.

### Loss of FABP3 Aggravates Chronic Overload-Induced Cardiac Hypertrophy

To examine the effects of FABP3 on cardiac hypertrophy, we generated F3-KO mice using CRISPR/Cas9, and these *Fabp3*-null mice were viable and fertile. Homozygous, heterozygous allele, and WT mice were identified using PCR (Supplementary Figures 1A–C). Notably, cardiac FABP3 was completely abolished using this knockout strategy, which allowed for a direct examination for FABP3 on cardiac hypertrophy (Supplementary Figures 1D,E).

To explore whether FABP3 contributes to TAC-induced hypertrophy, F3-KO and WT mice were subjected to TAC or sham surgery and observed for 4 weeks (Supplementary Figure 2A). Firstly, to exclude the systemic differences among TAC-operated WT and F3-KO mice, which may exert extra effects on the development of cardiac hypertrophy, the organ mass to body weight ratio was measured, and similar spleen, kidney, BAT, and white adipose tissue (WAT) ratio was found between WT and F3-KO mice (Supplementary Figure 2B). Similarly, no difference was observed in liver mass among WT and F3-KO mice (Supplementary Figure 2C). Moreover, hematoxylin and eosin (H&E) staining of the above organs showed similar tissue morphology and structure between WT and F3-KO mice after TAC surgery, demonstrating that FABP3 deletion did not result in systemic abnormity after TAC operations (Supplementary Figure 2D).

**Figure 2:**
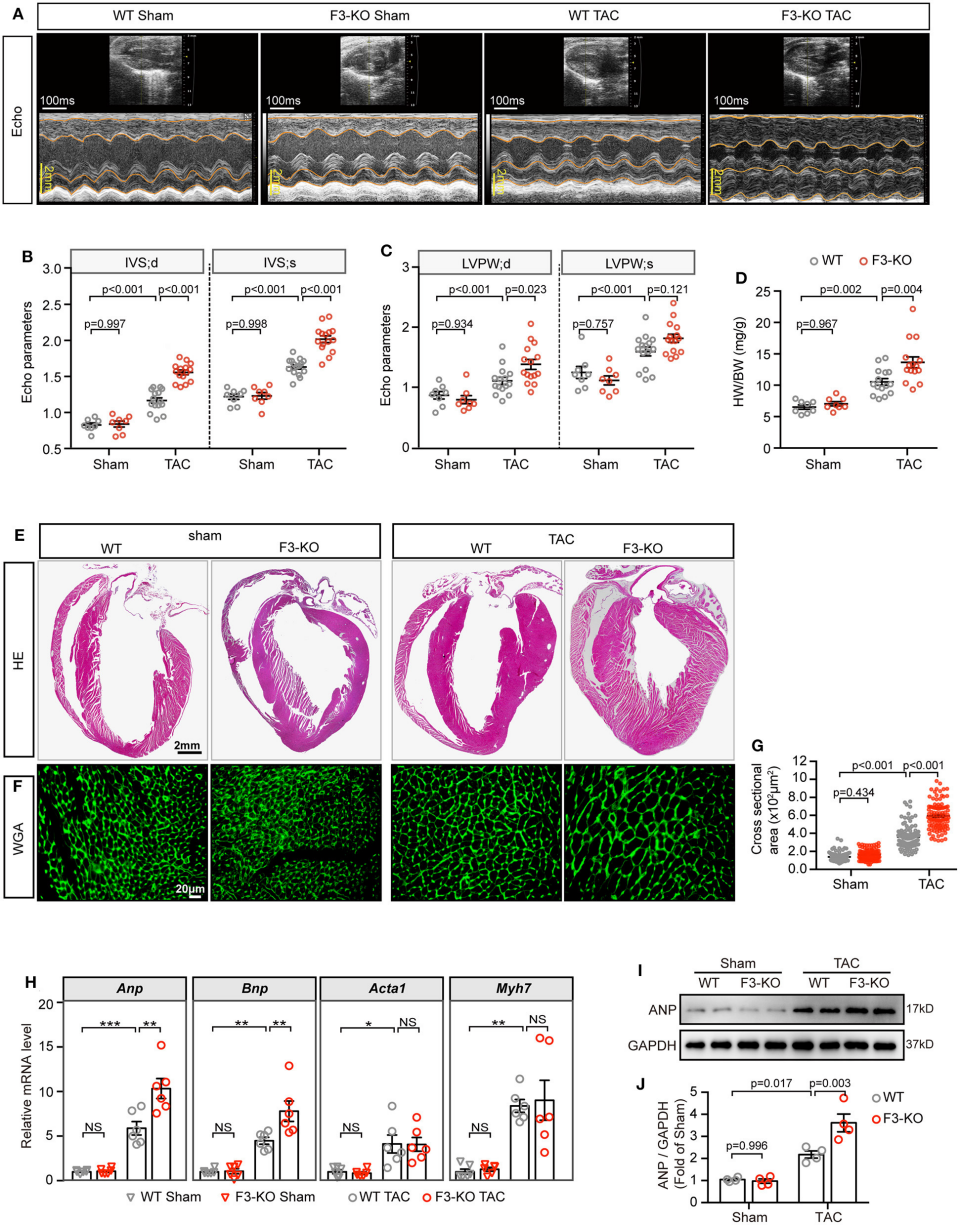
Deficiency of FABP3 aggravates TAC-induced cardiac hypertrophy. **(A)** Representative echo images of the WT and F3-KO mice at 4 weeks after TAC or sham operation. **(B)** Quantification results for the interventricular septum (IVS) in **(A)**. **(C)** Quantification results for the left ventricular posterior wall thickness (LVPW) in **(A)**. **(D)** The ratio of heart weight to body weight from sham or TAC-operated WT or F3-KO mice. **(E)** Images of H&E stained longitudinal sections of the indicated hearts. **(F)** Heart sections stained with WGA to compare the size of the cardiomyocyte area. **(G)** Quantification results in **(F)**; a total of 100 cells/group were calculated. **(H)** qPCR assays compared the mRNA expression of *Anp, Bnp, Acta1*, and *Myh7* at 4 weeks after sham or TAC operation. NS, not significant, *p < 0.05, **p < 0.01, ***p < 0.001. **(I)** Representative western blot images of ANP in the indicated groups. (J) Quantification results of **(I)**. [**B–D**, *n* = 8, 8, 15, 15, respectively; **H**, *n* = 6; **J**, *n* = 4; **(B–D,G,H,J)** Tukey's *post-hoc* test].

Next, to determine the effects of *Fabp3*-null on cardiac function, echocardiography (echo) was performed on WT and F3-KO mice at 4W after surgery (Figure 2A). Compared with the WT mice, TAC surgery led to severer cardiac hypertrophy in the *Fabp3-*null mice, revealed as higher thickness of the interventricular septum (IVS; d: 1.56 ± 0.03 vs. 1.17 ± 0.04; IVS; s: 2.02 ± 0.05 vs. 1.63 ± 0.03; F3-KO vs. WT) and left ventricular posterior wall thickness (LVPW; d: 1.38 ± 0.08 vs. 1.11 ± 0.06; LVPW; s: 1.82 ± 0.07 vs. 1.60 ± 0.07; F3-KO vs. WT) in the F3-KO mice; meanwhile, these parameters were comparable in sham groups (Figures 2B,C). Although no statistical difference was observed in the lung weight to body weight ratio (LW/BW) between the WT and KO mice (Supplementary Figure 2E), we found an increased heart weight to body weight ratio (HW/BW) in the F3-KO mice after TAC surgery compared with the WT mice (Figure 2D).

Then, histological H&E and WGA staining was conducted to determine the degree of cardiac hypertrophy after echo analysis. A significant increase in left ventricular wall thickness was found in F3-KO hearts compared to WT littermates after TAC surgery (Figure 2E). In addition, *Fabp3*-null caused a significantly enlarged cell area compared to the WT counterparts after TAC surgery (Figures 2F,G). In line with the above hypertrophic phenotype, the mRNA levels of *Anp* and *Bnp* were upregulated in F3-KO hearts after surgery compared to WT hearts (Figure 2H). Similarly, ANP levels were increased in F3-KO hearts compared to WT hearts (Figures 2I,J). Altogether, we concluded that FABP3 deficiency promoted cardiac hypertrophy after TAC operations.

### Loss of FABP3 Impairs Cardiac Remodeling After Hypertrophy

Cardiac hypertrophy contributes to heart failure under chronic overload, ultimately leading to adverse cardiovascular events and death. To determine whether *Fabp3* deficiency is associated with heart failure, the WT and F3-KO mice were observed over 8 weeks after TAC surgery. At the end of the observation period, a lower survival probability was observed in the F3-KO group compared to the WT group (Supplementary Figure 3A, *p* = 0.3), which was accompanied with increased heart size in the F3-KO mice after TAC surgery compared to the WT hearts (Supplementary Figure 3B). Consistent with the enlarged heart size in the *Fabp3*-defect mice, the decline in left ventricular ejection fraction (LVEF) and left ventricular fractional shortening (LVFS) was found in the F3-KO mice compared to their WT group at 8 weeks post-surgery, which was paralleled with higher left ventricular end diastolic volume (LVEDV) and left ventricular end systolic volume (LVESV) in the *Fabp3*-null mice (Supplementary Figures 3C–E).

Then, considering that fibrosis serves as a hallmark of cardiac dysfunction, by measuring the level of fibrosis-related genes via qPCR assay, we found that the mRNA expression of *Col3a1* was increased in the TAC-operated F3-KO hearts compared to the WT hearts. Moreover, *Fabp3* ablation reduced the mRNA expression of matrix metallopeptidase 2 (*Mmp2*) and matrix metallopeptidase (*Mmp9*) after TAC surgery, suggesting impaired collagen turnover and homeostasis in the *Fabp3*-KO mice (Supplementary Figure 3F). In agreement with the increased expression of fibrosis genes in the F3-KO hearts, Massons and Sirus red staining confirmed increased left ventricular collagen volume in the F3-KO hearts compared to the WT hearts (Supplementary Figures 3G,H). Cumulatively, these data suggest that the loss of FABP3 contributes to the progression of heart failure following TAC operation.

### FABP3 Alleviates Ang II-Induced Cardiomyocyte Hypertrophy *in vitro*

**Figure 3:**
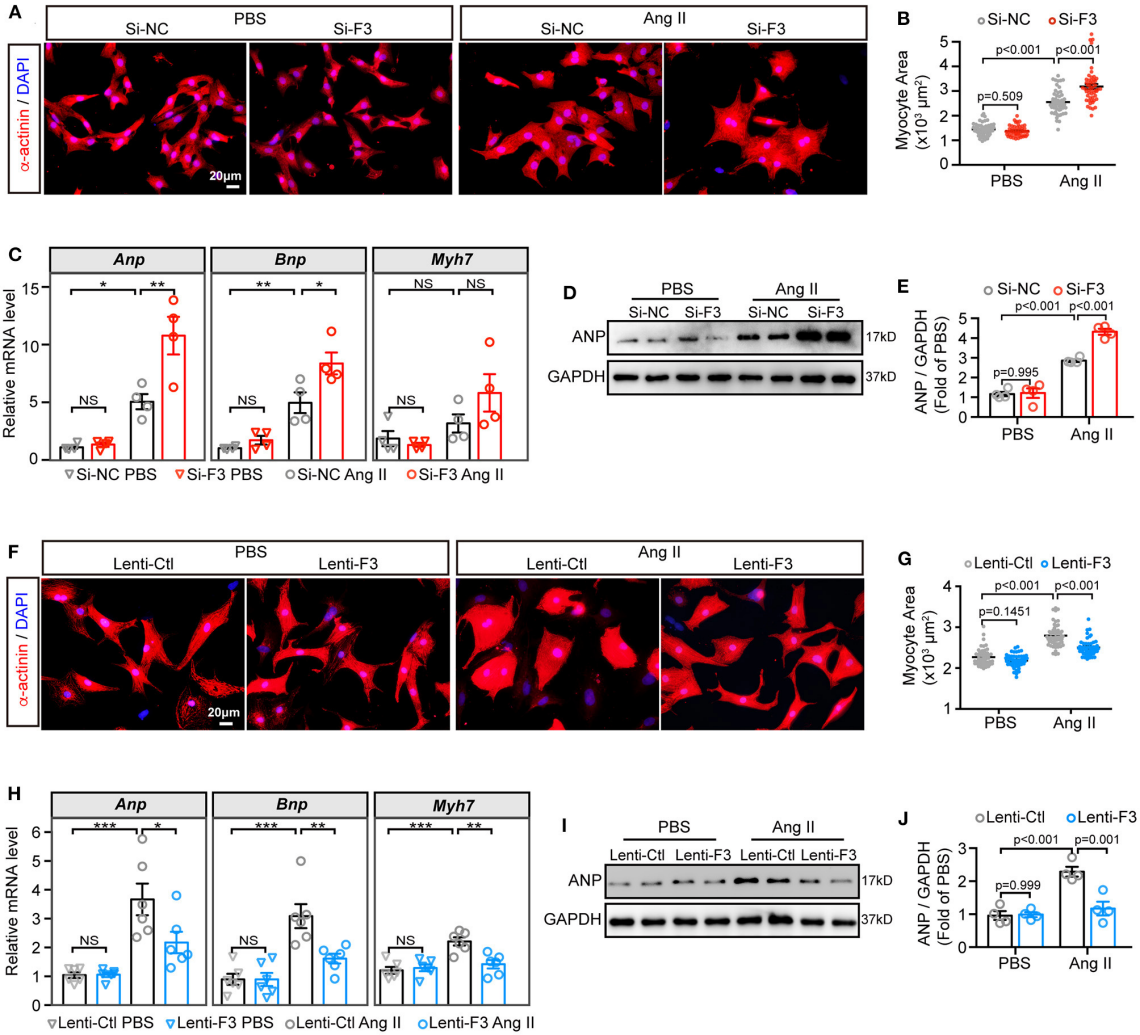
FABP3 participates in Ang II induced cell hypertrophy. **(A–E)** NRVMs were treated with siRNA-targeting *Fabp3* (Si-F3) or its negative control (Si-NC) before PBS or Ang II treatment. **(A)** Immunofluorescence staining of α-actinin in NRVMs with or without Ang II treatment. **(B)** Quantification results of the cell area in (A); 50 cells/group were calculated. **(C)** mRNA expression of hypertrophic genes (*Anp, Bnp*, and *Myh7*) in indicated groups. NS, not significant, ^*^p < 0.05, **p < 0.01. **(D)** Representative western blot images of ANP after *Fabp3* knocking-down. **(E)** Quantification of **(D)**. **(F–J)** NRVMs were transfected with lentivirus-encoding *Fabp3* (Lenti-F3) or its negative control (Lenti-Ctl) before PBS or Ang II treatment. **(F)** The cell area was determined by α-actinin staining in NRVMs. **(G)** Quantification results of the cell area in **(F)**; 50 cells/group were calculated. **(H)** The mRNA levels of *Anp, Bnp*, and *Myh7* were compared in the aforementioned groups. NS, not significant, *p < 0.05, **p < 0.01, ***p < 0.001. **(I)** Representative western blot images revealed reduced ANP protein levels in the Lenti-F3 group. **(J)** Quantification results of **(I)**. [**C,E,J**, *n* = 4; H, *n* = 6; **(B,G)**: Games–Howell *post-hoc* test; **(C,E,H,J)**: Tukey's *post-hoc* test].

To corroborate the above findings that FABP3 participates in TAC-induced hypertrophy, we knocked down the expression of *Fabp3 in vitro* using non-targeting small interfering RNA (siRNA) (Supplementary Figure 4A). The cell size was comparable in the PBS groups; however, knocking down *Fabp3* significantly enlarged the cell area after Ang II treatment compared with the Si-NC group (Figures 3A,B). Moreover, an increase in the *Anp* and *Bnp* mRNA expression in line with the upregulation of the ANP protein level was found in the Si-F3 group compared with the Si-NC group (Figures 3C–E).

Next, lentivirus vectors containing the full-length *Fabp3* transcript (NM_001320996) and green fluorescent protein (GFP) were constructed, transfected, and visualized with the fluorescence microscope to confirmed the transfection efficiency (Supplementary Figure 4B). After transfecting H9C2 cells with optimal multiplicity of infection (MOI) of 10 and 100, lentivirus carrying the *Fabp3* transcript (Lenti-F3) markedly increased FABP3 protein levels by more than four-fold compared to the empty control vector (Lenti-Ctl; Supplementary Figures 4C,D), which was supported by a higher FABP3 fluorescence value in the Lenti-F3 group (Supplementary Figure 4E). In contrast to the pro-hypertrophic effect of *Fabp3* ablation, the knocking-in expression of FABP3 prevented Ang II-induced cell hypertrophy *in vitro*. Specifically, Ang II stimulation resulted in enlarged cell sizes in the control group, which was markedly blunted with the overexpression of FABP3 (Figures 3F,G). Consistent with the reduction in the cell area, the overexpression of FABP3 curbed the increase in *Anp, Bnp*, and *Myh7* mRNA levels following Ang II treatment (Figure 3H), as well as the protein expression of ANP (Figures 3I,J). These *in vitro* data verified the protective effects of FABP3 on neurohormonal stimuli-induced hypertrophy. Altogether, these FABP3 loss- and gain-of-function results corroborate with *in vivo* phenotypes and reveal the important role of FABP3 in the development of cardiac hypertrophy.

### Ablation of FABP3 Leads to Defective FA β-Oxidation and Lipid Homeostasis

**Figure 4:**
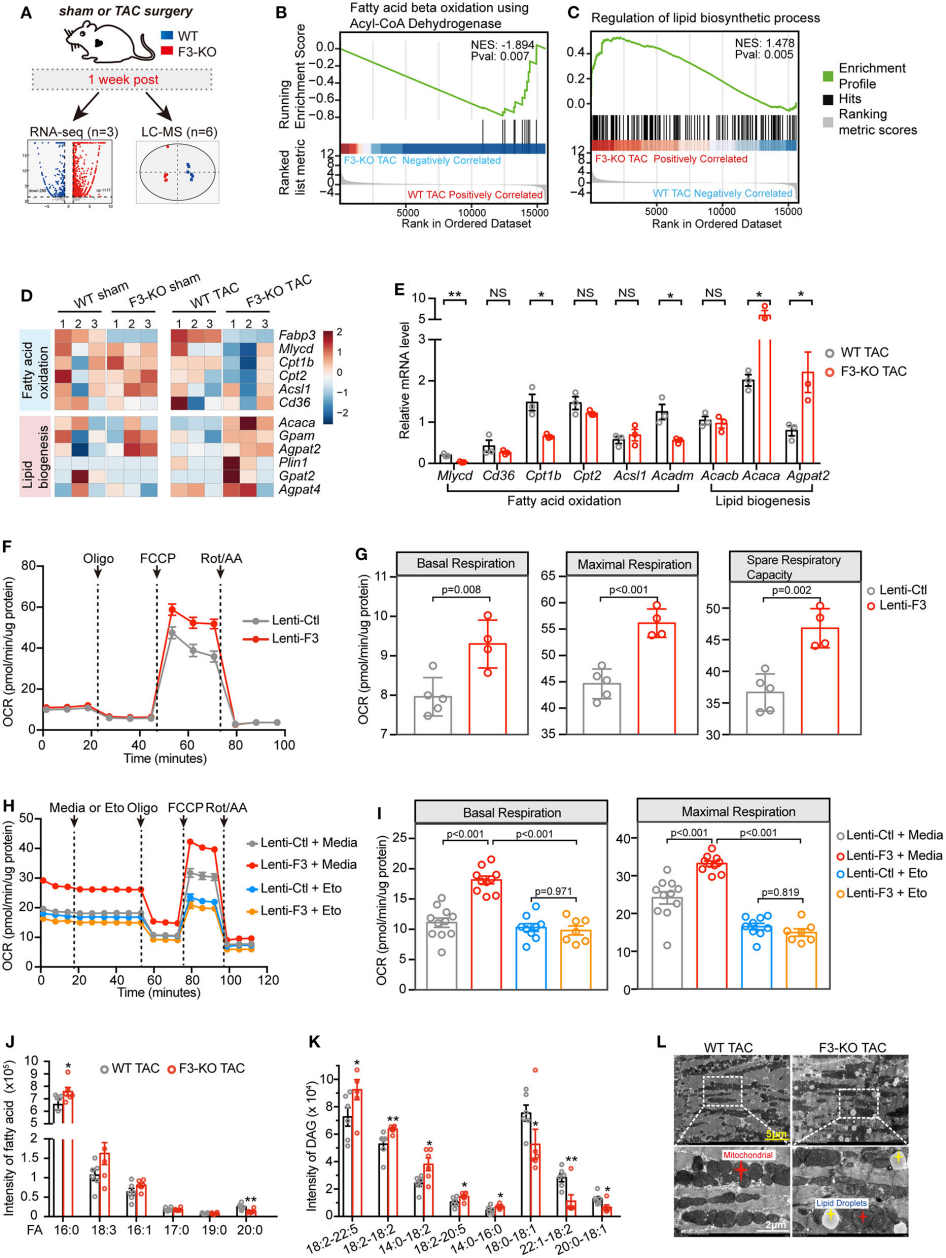
FABP3-defect results to compromised fatty acid oxidation (FAO) and toxic lipid accumulation. **(A)** Experimental schematic of RNA-seq analysis and metabolomics analysis in WT and F3-KO hearts. **(B,C)** GSEA revealing that F3-KO negatively correlates with fatty acid beta oxidation using acyl-CoA dehydrogenase **(B)**, while positively correlating with the lipid biosynthetic process **(C)**. **(D)** Heatmap showed scaled expression of FAO and lipid biogenesis genes in indicated samples from RNA-seq analysis. **(E)** The mRNA expression of FAO and lipid biogenesis genes in TAC-operated WT and F3-KO hearts. NS, not significant, *p < 0.05, **p < 0.01. **(F)** Mitochondrial stress assay was performed in NRVMs transfected with *Fabp3* (Lenti-F3) or its negative control (Lenti-Ctl) after Ang II treatment to measure the oxygen consumption rates (OCR); data were presented as mean ± SD from individual experiments. **(G)** The parameters of basal respiration, maximal respiration, and spare respiration capacity were calculated from **(F)**; data were presented as mean ± SD from individual experiments. **(H)** LCFA oxidation stress assay was conducted in NRVMs with or without knocking-in expression of *Fabp3* after Ang II treatment; etomoxir (Eto) was applied to inhibit the mitochondrial FAO. Data were presented as mean ± SEM from individual experiments. **(I)** The parameters of basal respiration and maximal respiration were calculated from **(H)**; data were presented as mean ± SEM from individual experiments. (J and K) The level of fatty acid (FA) and diacylglycerol (DAG) in WT and F3-KO hearts determined by LC-MS analysis; six biological replicates per group. *p < 0.05, **p < 0.01. **(L)** Representative electron micrographs of WT and F3-KO hearts after TAC operation. (Bottom) Higher magnification images of the dashed rectangle from **(L)**. [**E**, *n* = 3, Student's *t*-test; **G**, *n* = 5, 4, respectively, Student's *t*-test; **I**, *n* = 11, 10, 9, 7, respectively, Tukey's *post-hoc* test; **(J,K)** Student's *t*-test].

To explore the mechanism through which FABP3 regulates cardiac hypertrophy, we collected F3-KO or WT hearts at 1-week post-sham or -TAC operation for RNA-seq analysis and liquid chromatography–mass spectrometry (LC-MS) analysis to determine differential genes and metabolites (Figure 4A). Principal component analysis (PCA) of RNA-seq revealed that the transcriptome of the F3-KO hearts was similar to those of the WT mice under sham conditions. However, TAC operation induced differentially expressed genes between WT and F3-KO hearts, which separated them on the PCA plot (Supplementary Figure 5A). Comparing the TAC-operated F3-KO hearts with the WT hearts, a total of 939 (upregulated: 772, downregulated: 167) differentially expressed genes were identified and analyzed in this study (Figure 4A). KEGG pathway analysis revealed that these differentially expressed genes were enriched for terms related to “lipid metabolism,” “glycan metabolism,” and “energy metabolism” (Supplementary Figure 5B). Next, gene set enrichment analysis (GSEA) revealed that pathways that involved “regulation of anatomical structure size,” “wound healing,” and “extracellular structure organization” were positively correlated with the TAC-operated F3-KO mice. These pathways, which were consistent with pro-hypertrophic phenotypes in the *Fabp3*-null hearts, suggested that ablation of *Fabp3* triggers maladaptive remodeling after TAC operation (Supplementary Figure 5C).

Intriguingly, GSEA indicated that *Fabp3* ablation resulted in impaired mitochondrial FAO and disrupted lipid homeostasis. Specifically, “fatty acid beta oxidation using acyl-CoA dehydrogenase” was negatively correlated with the F3-KO hearts (Figure 4B; Supplementary Figure 5C), while positively enriched “lipid biosynthetic process” and “lipid storage” were found in the F3-KO hearts compared with the WT hearts (Figure 4C; Supplementary Figure 5D). In line with the aforementioned pathways, suppressed FAO genes and upregulated lipid biogenesis genes were observed in the F3-KO TAC group than the WT TAC group in RNA-seq analysis and *in vivo* experiments (Figures 4D,E). However, these genes were comparable under sham conditions whether in RNA-seq analysis or their mRNA expression *in vivo* (Figure 4D; Supplementary Figure 5E), which manifested that *Fabp3* deficiency has no profound effect on cardiac lipid metabolism under physiological conditions; however, hypertrophic stimulation that occurred with higher energy demand magnified the effects of *Fabp3*-defect on energy homeostasis and resulted in imbalanced FAO and lipid biosynthesis processes.

Furthermore, to explore the effects of FABP3 on cardiomyocyte FAO activity under hypertrophic stimulation *in vitro*, mitochondrial stress assay was performed in NRVMs with or without the knocking-in expression of FABP3 to measure in real time the oxygen consumption rate (OCR). Compared with the negative control, overexpression of FABP3 resulted in significant increases in basal, maximal respiration, and spare respiratory capacity (Figures 4F,G). Relative to glucose, fatty acid required more oxygen for its β-oxidation; therefore, the OCR serves as a relative indicator of cell fuel preference. The higher OCR rate in the lenti-F3 group indicated that the overexpression of FABP3 resulted in the increase in FAO activity. Then, a supplement of etomoxir was used to evaluate the cell dependency on fatty acid as the energy substrate. We found that the application of etomoxir led to a marked drop of respiration in the FABP3 overexpressed group compared with its negative control groups, demonstrating that NRVMs with FABP3 overexpression have increased reliance on fatty acid as energy fuel (Figures 4H,I). Moreover, etomoxir treatment would reverse the protective effects of FABP3 on cardiac hypertrophy and upregulated the mRNA expression of *Bnp* and *Anp* (Supplementary Figure 5F). Taken together, these data point to the dependency of FABP3 on cardiomyocyte FA β-oxidation to meet an effective metabolic demand under hypertrophic stimulations.

### FABP3-Null Hearts Exhibit Abnormal Lipid Accumulation

The RNA-seq analysis showed that FABP3 deletion led to increased lipid biogenesis (Figures 4C,D). Lipid-targeted metabolomics analysis was used to determine the differential metabolites in WT and F3-KO hearts. We found that rather than triglycerides (TAG, Supplementary Figure 6B) and Acyl-carnitine (ACar, Supplementary Figure 6C), the fatty acid (16:0) and diglyceride (DAG 18:2–22:5, 18:2–18:2) were significantly increased in the F3-KO hearts compared with the WT mice (Figures 4J,K), which was in line with an increase in neutral lipid in *Fabp3* knocking-down cells after Ang II treatment (Supplementary Figure 6A). It is important to note that DGA and saturated fatty acid (especially palmitate) were closely associated with cellular toxicity for their direct actions as signaling lipids (18). Therefore, FABP3 deficiency not only blunted cardiac FA β-oxidation but also triggered the excessive accumulation of toxic lipids in hearts. Consistent with increased toxic lipid species and neutral lipid *in vivo* and *in vitro*, an excess of lipid droplet accumulation was observed in the *Fabp3*-null hearts after TAC surgery by using transmission electron microscopy (TEM), which was rare in the WT hearts (Figure 4L; Supplementary Figure 6D). To determine whether differences in lipid uptake accounted for the abnormal lipid accumulation in the F3-KO mice, plasma non-esterified fatty acid (NEFA) was measured at sham, 4W and 8W post-surgery; we found that the plasma concentration of NEFA was higher at 8W compared to the sham group; however, no significant difference was observed between WT and F3-KO mice (Supplementary Figure 6E). These data in line with comparable mRNA expression of fatty acid transporter *Cd36* between WT and F3-KO hearts (Figures 4D,E) revealed that abnormal lipid accumulation in the F3-KO hearts may arise from activated lipid biosynthesis rather than abnormal lipid uptake. Taken together, we showed that *Fabp3*-null contributes to defective FAO and increased lipid biogenesis and toxic lipid accumulation after cardiac hypertrophy (Supplementary Figure 6F).

### FABP3 Defect Hearts Show Increased Reliance on Glycolysis

**Figure 5:**
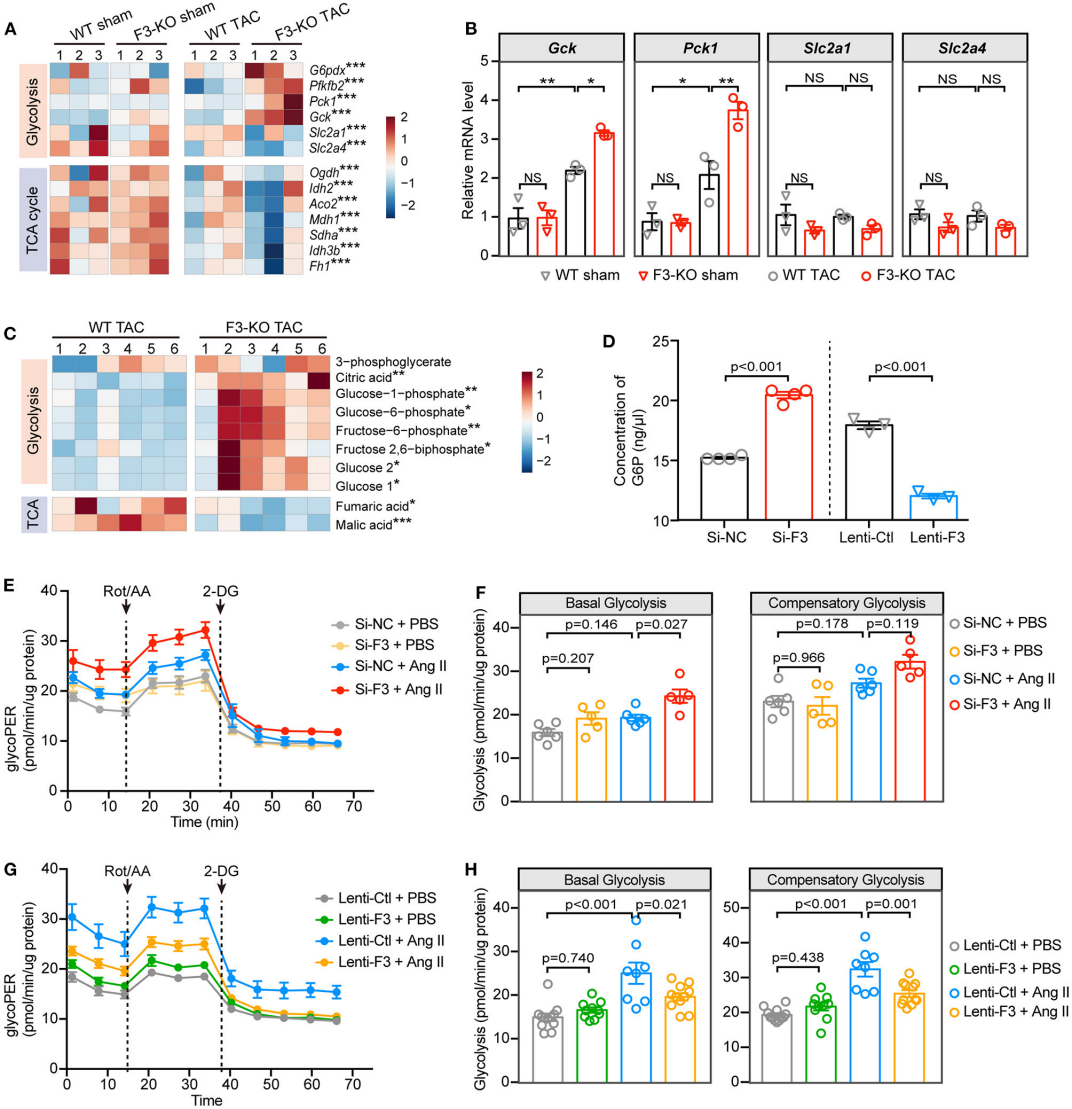
FABP3-null contributes to increased glycolysis and reduced ATP production under hypertrophic stimulation. **(A)** Heatmap of glycolysis and TCA cycle genes from RNA-seq analysis. **(B)** The mRNA expression of *Gck, Pck1, Slc2a1*, and *Slc2a4* in indicated groups. NS, not significant, *p < 0.05, **p < 0.01. **(C)** Heatmap of differential metabolites from WT and F3-KO hearts. *p < 0.05, **p < 0.01, ***p < 0.001. **(D)** The intracellular concentration of glucose-6-phosphate (G6P) in NRVMs was measured after manipulating the expression of *Fabp3*. **(E)** Glycolytic rate assay was performed in NRVMs after knocking down the expression of *Fabp3* with siRNA to measure the OCR and extracellular acidification rates (ECAR) and converted to glycolytic proton efflux rate (glycoPER) in the Seahorse Report Generator. **(F)** The parameters of basal glycolysis and compensatory glycolysis were calculated from **(E)**. Data were presented as mean ± SEM from individual experiments. **(G)** Glycolytic rate assay was performed in NRVMs with or without *Fabp3* overexpression to measure glycoPER. **(H)** The parameters of basal glycolysis and compensatory glycolysis were calculated from **(G)**. Data were presented as mean ± *SD* from individual experiments. [**B**, *n* = 3, Tukey's *post-hoc* test; **D**, *n* = 4, 4, 3, 3, respectively, Student's *t*-test; **F**, *n* = 6, 5, 6, 5, respectively; **H**, *n* = 12, 10, 8, 11, respectively; **(F,H)** Tukey's *post-hoc* test].

In the context of diminished capacity for FAO, we next sorted to determine whether the chief energy substrate shifted from fatty acids to glucose when FABP3 is defected. Intriguingly, GSEA revealed that the loss of FABP3 triggered abnormally activated glucose metabolic pathways, such as “glucan catabolic process” (Supplementary Figure 7A) and “regulation of gluconeogenesis” (Supplementary Figure 7B). In line with activated glucose oxidation and gluconeogenesis pathways, *Gck, Pfkfb2*, and *Pck1* were upregulated in the F3-KO TAC hearts (Figure 5A). A consistent mRNA expression profile *in vivo* confirmed the increased *Gck* and *Pck1* level in the *Fabp3* deficiency hearts after TAC surgery (Figure 5B). Intriguingly, “mitochondrial electron transport NADH to ubiquinone” was negatively correlated with the F3-KO hearts, which was consistent with markedly downregulated TCA cycle genes (*Ogdh, Idh2, Aco2*) in TAC-operated *Fabp3*-null hearts than in the WT mice (Figure 5A; Supplementary Figure 7C). These results indicated that rather than fuel-efficient aerobic respiration, the *Fabp3*-null hearts shifted toward inefficient anaerobic respiration (glycolysis) for ATP production. As increased glucose uptake would lead to higher glucose oxidation, we interrogated the expression glucose transporters (Slc2a1, Slc2a4), the concentration of blood glucose, and the glycogen content in liver and small intestine, and the results showed that the glucose uptake was comparable between the WT and F3-KO mice during cardiac hypertrophy (Figures 5A,B; Supplementary Figures 7D,E).

Furthermore, the gas chromatography–mass spectrometry (GC-MS) analysis was used to identify differential metabolites in WT and F3-KO hearts after TAC surgery. Principal component analysis showed that the F3-KO hearts were separated from the WT samples, which was consistent with the OPLS-DA analysis results (Supplementary Figures 7F–H). Notably, significantly increased glycolysis pathway related metabolites [glucose, glucose-6-phosphate (G6P), and fructose 2,6-biphosphate] and considerably downregulated TCA cycle metabolites (malic and fumaric acids) were observed in the *Fabp3*-deficient hearts as compared to the WT hearts (Figure 5C). Furthermore, by measuring the concentration of G6P, an indicator of cellular glycolytic flux, we showed a higher concentration of G6P in *Fabp3* knocking-down cells; however, overexpression of *Fabp3* resulted in dramatic decline in the G6P level (Figure 5D). These data confirmed that FABP3 defect contributed to the increased glycolysis under hypertrophic conditions.

Based on the results from the RNA-seq and metabolomics, we performed a glycolytic rate assay to analyze in real time the cellular glycolysis by calculating the proton efflux rate from glycolysis (glycoPER), a parameter that measures acidification from glycolysis without any contribution from mitochondrial respiration. NRVMs with *Fabp3* knocking-down exhibited a higher basal and compensatory glycolysis compared to its negative control after Ang II stimulation; however, the glycolytic rate showed no significant differences between these two groups under PBS treatment (Figures 5E,F). In striking contrast to increased glycolysis when knocking down the expression of *Fabp3, Fabp3* overexpression resulted in a marked drop of glycolysis than the control cells after Ang II treatment (Figures 5G,H). By combining with multi-omics analysis and glycolytic energetics analysis, we demonstrated that *Fabp3* deficiency promoted the shift on glycolysis as the fuel source, which led to compromised TCA and ATP production (Supplementary Figure 7I). Taken together, these observations confirm that FABP3 defect contributes to compromised FAO and ATP production; on the other hand, it exacerbates glycolysis and toxic lipid accumulation, both of which ultimately aggravate metabolic derangement and heart failure.

### FABP3 Mediates PPARα Level by Binding and Stabilizing PPARα, Further Enhances Its Transcriptional Activity Under Hypertrophic Stimuli

**Figure 6:**
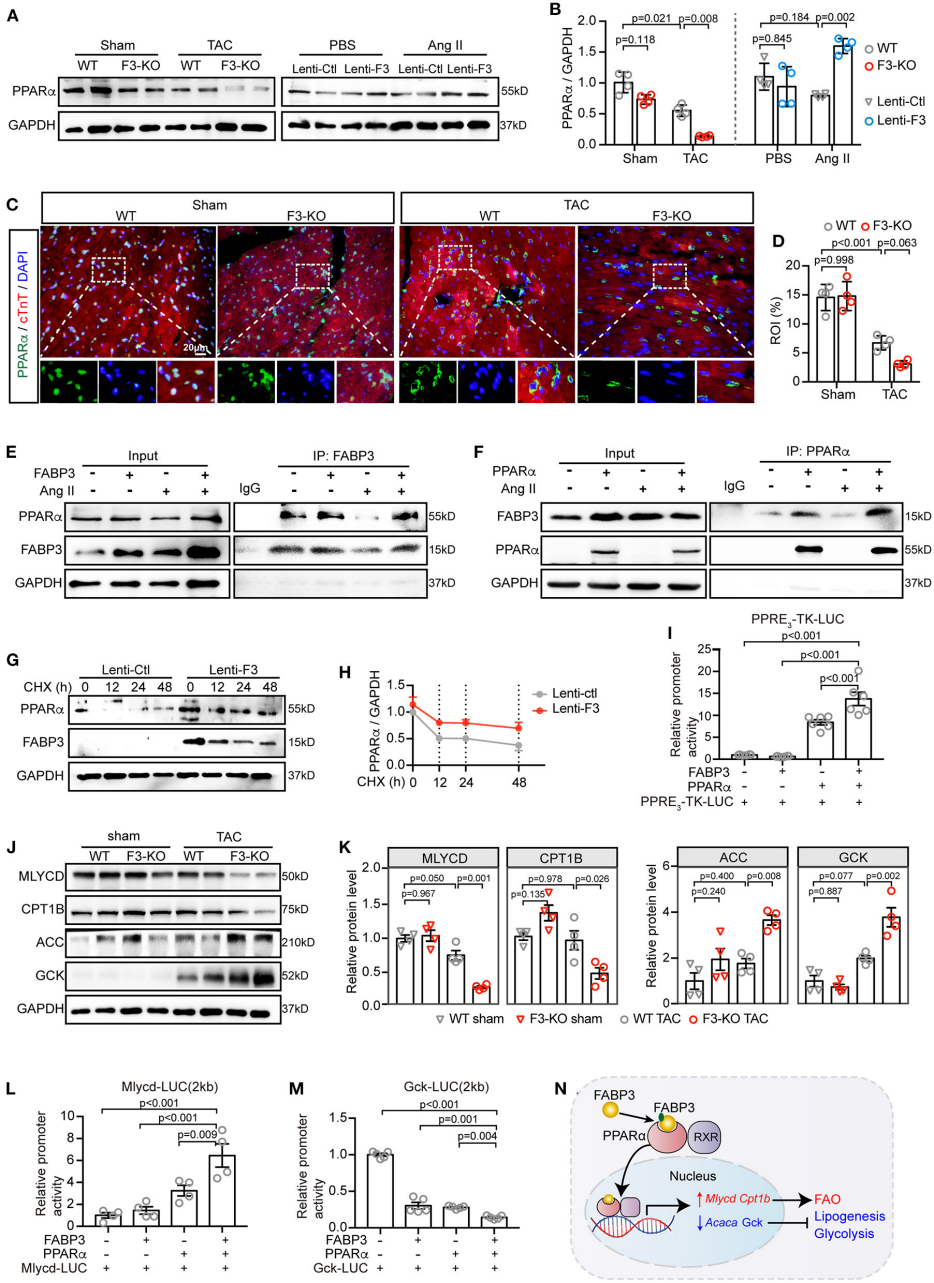
FABP3 mediates metabolic reprogramming by directly interacting with PPARα, preventing its degradation and enhancing its transactivation. **(A)** Representative western blot images of PPARα from TAC-operated WT and F3-KO hearts, or from NRVMs transfected with *Fabp3* or its control virus with or without Ang II treatment. **(B)** Quantification of **(A)**. **(C)** Immunofluorescence double-staining of PPARα (green) and cTnT (red) in WT and F3-KO hearts, with or without TAC surgery. (Bottom) Higher magnification of dashed rectangle in the upper panel. **(D)** Quantification results in **(C)**. **(E)** NRVMs with *Fabp3* overexpression were treated with or without Ang II for 24 h and then co-immunoprecipitated (co-IP) with FABP3. Western blotting assay was conducted with indicated antibodies. **(F)** NRVMs with *Ppar*α overexpression were treated with or without Ang II for 24 h and then co-immunoprecipitated (co-IP) with PPARα. **(G)** NRVMs transfected with Lenti-F3 or Lenti-Ctl were treated with CHX for 0, 12, 24, or 48 h and the protein expression of PPARα was measured by western blotting. **(H)** Quantification of PPARα intensities by normalizing to those of GAPDH in **(G)**. **(I)** HEK 293T cells were transfected with a PPRE-driven luciferase reporter (PPRE_3_-TK-LUC) and PPARα or FABP3 for 24 h. Relative activation of PPRE_3_-TK-LUC was measured by normalizing its luminescence value to the renilla activity. **(J)** Representative western blot images of MLYCD, CPT1B, ACC, and GCK in WT and F3-KO mice after sham or TAC operation. **(K)** The quantification results of **(J)**. **(L,M)** HEK 293T cells were transfected with a *Mlycd*-promoter luciferase reporter (Mlycd-LUC, **L**) or a *Gck*-promoter luciferase reporter (Gck-Luc, **M**), PPARα, or FABP3 for 24 h. The relative expression of the *Mlycd*-promoter and *Gck*-promoter was measured by normalizing their luminescence value to the corresponding renilla luminescence value. **(N)** The schematic diagram shows FABP3 binds to PPARα and increases its transcriptional activity on *Mlycd* and *Cpt1b* while repressing *Acaca* and *Gck* to participate in cardiac FAO/glycolysis shift. [**B**, *n* = 4, Games–Howell *post-hoc* test; **D**, *n* = 4, Tukey's *post-hoc* test; **H**, *n* = 2; I, *n* = 6, Dunnett's *post-hoc* test; **K**, *n* = 4, Tukey's *post-hoc* test; **L**, *n* = 4; **M**, *n* = 5; (**L,M**) Dunnett's *post-hoc* test].

As mentioned before, *Fabp3* deficiency contributes to deranged metabolic milieu characterized by reduced FAO and increased glycolysis. Next, we sought to explore the mechanism through which *Fabp3* mediates metabolic derangement during cardiac hypertrophy. Firstly, our RNA-seq analysis verified that the “PPAR signaling pathway” was one of the top enriched pathways in the *Fabp3*-deficient hearts (Supplementary Figure 8A). Definitive evidence demonstrates the critical requirement of peroxisome proliferator activated receptor (PPAR), particularly PPARα, in myocyte metabolism and metabolic reprogramming under cardiac hypertrophy (19). Therefore, we postulated that FABP3 participates in cellular metabolism through the PPARα signaling pathway. Although the mRNA expression of *Ppar*α showed no difference in WT and F3-KO hearts (Supplementary Figure 8B), its protein expression was marked downregulation in the *Fabp3* deficiency mice as compared to the WT hearts following TAC surgery (Figures 6A,B). Conversely, *in vitro* overexpression of *Fabp3* using a lentivirus vector rescued the protein level of PPARα following Ang II stimulation (Figures 6A,B). Immunofluorescence staining of PPARα at 4W post-surgery confirmed that TAC operation resulted in the decrease and perinuclear shuttling of PPARα, while *Fabp3* ablation accelerated its loss (Figures 6C,D). These findings indicate that FABP3 might participate in metabolic homeostasis during cardiac hypertrophy via PPARα signaling. However, the mechanism through which FABP3 targets PPARα for metabolic regulation remains elusive.

Next, by transfecting NRVMs with *Fabp3* or *Ppar*α and co-immunoprecipitating with respective antibodies, we showed that FABP3 directly bond with PPARα, with or without Ang II stimulation (Figures 6E,F). As *Fabp3* deletion exerted no effect on the *Ppar*α mRNA level, we postulated that FABP3 exerted a post-transcriptional modification on the protein level of PPARα. Expectedly, *Fabp3* overexpression markedly prolonged the half-life of PPARα compared with its negative control, suggesting that FABP3 increased the PPARα protein level by inhibiting its degradation (Figures 6G,H). Finally, to determine whether FABP3 affected PPARα transactivation, we performed luciferase gene transactivation assays in HEK 293T cells. The activation of PPARα was determined based on a reporter plasmid containing three PPAR-responsive elements (PPRE_3_-TK-LUC). PPARα significantly increased the luciferase expression of PPRE_3_-TK-LUC; moreover, cotransfection of FABP3 and PPARα induced higher PPRE-driven luciferase activity compared with PPARα alone (Figure 6I). Together, these findings indicate that FABP3 mediates hypertrophic response by interacting with PPARα, inhibiting its degradation, and modulating its transcriptional activity.

### Required of PPARα on FABP3-Modulated FAO/Glycolysis Balance and Cardiac Hypertrophy

As regard the pleiotropic effects of PPARα on inhibiting glucose oxidation, while activating FAO, we aimed to determine whether FABP3 interacts with PPARα and modulates its transcriptional capacities on FAO/glycolysis genes, and further is involved in the advance of cardiac hypertrophy. Firstly, the PPARα target genes were curated based on our RNA-seq data and listed as Supplementary Table 1. In line with previous results in Figures 4, 5, *Fabp3* ablation led to a lower MLYCD and CPT1B protein level while upregulating ACC and GCK, suggesting a direct effect of FABP3 on MLYCD and CPT1B, and an inverse transcriptional effect of FABP3 on ACC and GCK via PPARα (Figures 6J,K). Therefore, to address the question of whether FABP3 participated in the transcriptional activation of FAO and glycolysis genes via PPARα, the plasmid containing firefly luciferase and *Mlycd* or *Gck* promoter (Mlycd-LUC, Gck-LUC, respectively) was constructed; after transfecting 293T cells with PPARα, FABP3, Mlycd-LUC, or Gck-LUC, we showed that PPARα increased the *Mlycd* transcriptional activity. However, higher luciferase activity was observed when it was cotransfected with FABP3 and PPARα (Figure 6L). In contrast to Mlycd-LUC, PPARα blunted the transactivation of Gck-LUC, which showed a severer inhibition in the presence of FABP3 (Figure 6M). These data suggest the metabolic regulatory role of FABP3 in transcriptionally activating FAO genes *Mlycd* and *Cpt1b* and curbing glycolysis and lipogenesis genes *Gck* and *Acaca* via PPARα (Figure 6N).

Finally, to demonstrate the requirement of PPARα in FABP3 mediated cardiac hypertrophy, PPARα was knocked down using siRNA methods as described previously (Supplementary Figures 8C,D) (20). We observed that PPARα downregulation abolished the protective effects of FABP3 on cardiomyocyte hypertrophy, resulting in an enlarged cell area (Supplementary Figures 8E,F) and the upregulation of *Bnp* and *Anp* (Supplementary Figure 8G). Notably, increased cellular neutral lipid was found after knocking down PPARα (Supplementary Figure 8H). Altogether, these data illustrate that FABP3 participates in cardiac hypertrophy by synergistically activating *Mlycd* and *Cpt1b* and repressing *Gck* and *Acaca* via PPARα.

### Activation of PPARα With Fenofibrate Reverses FABP3-KO Induced Cardiac Hypertrophy

**Figure 7:**
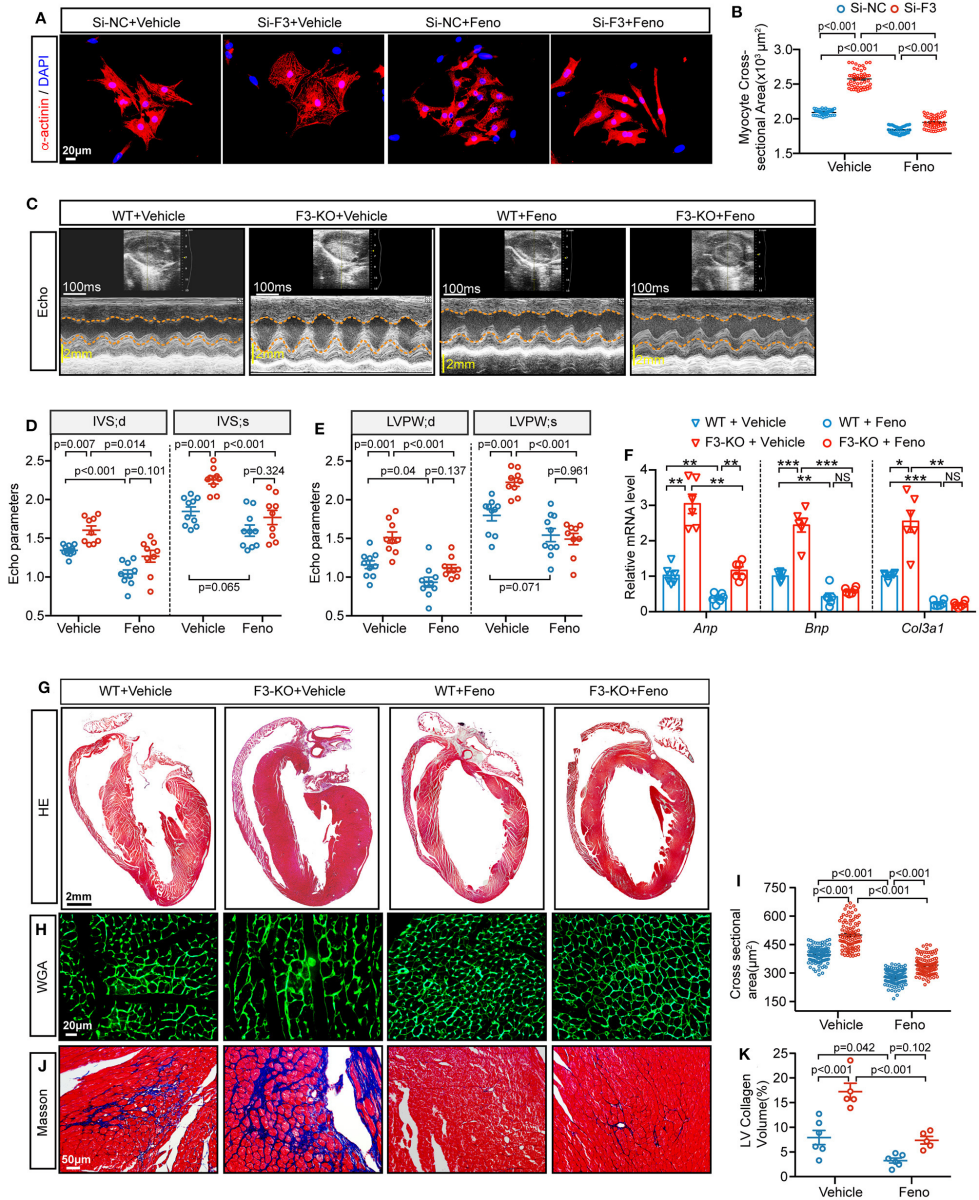
Fenofibrate represses FABP3-null induced cardiac hypertrophy *in vivo* and *in vitro*. **(A)** NRVMs were treated with siRNA targeted *Fabp3* (Si-F3) or its negative control before fenofibrate or vehicle treatment. Immunofluorescence staining of α-actinin used to compare the myocyte area. **(B)** Quantification of **(A)**. **(C)** Representative echo images in WT or F3-KO mice with or without fenofibrate treatment (Feno or Vehicle, respectively) at 4 weeks after TAC surgery. **(D)** Quantification of IVS in **(C)**. **(E)** Quantification of LVPW in **(C)**. **(F)** Relative mRNA levels of *Anp, Bnp*, and *Col3a1*, as determined by qPCR in the indicated groups. NS, not significant, *p < 0.05, **p < 0.01, ***p < 0.001. **(G)** Images of H&E-stained longitudinal heart sections from WT or F3-KO mice with or without fenofibrate treatment. **(H)** Representative immunofluorescence images of WGA staining from WT or F3-KO mice with or without fenofibrate treatment. **(I)** Quantification of the cardiomyocyte cross-sectional area in **(I)** (*n* = 100). **(J)** Masson staining to determine cardiac fibrosis in the aforementioned groups. **(K)** Quantification of collagen volume in **(L)**. [**D, E**, *n* = 10, 9, 10, 9, respectively; (**B**, D-IVS; s): Games–Howell *post-hoc* test; (D-IVS; d, **E**): Tukey's *post-hoc* test; **F**, *n* = 6, (F-Anp, F-Col3a1, and **I**): Games–Howell *post-hoc* test; **K**, *n* = 6, 5, 6, 5; (F-Bnp, and **K**): Tukey's *post-hoc* test].

As observed previously, *Fabp3* deficiency contributes to hypertrophy and deranged metabolic milieu by impairing the PPARα pathway. We next sought to determine whether activating PPARα may rescue the pro-hypertrophic effects of *Fabp3* defect after TAC operation and search for clinical benefits on the treatment of cardiac hypertrophy. Firstly, NRVMs were treated with fenofibrate, a PPARα-specific agonist, or vehicle (DMSO) for 24 h; knocking down of *Fabp3* with siRNA (Si-F3) resulted in an increased cell area compared to its control (Si-NC), while fenofibrate treatment markedly reduced the cell area in both the Si-NC and Si-F3 groups (Figures 7A,B).

Next, PPAR agonist studies were performed *in vivo* to investigate the effects of fenofibrate on hypertrophy. WT and *Fabp3*-KO mice were subjected to TAC surgery and randomly treated with fenofibrate (100 mg/kg/day) or vehicle by oral gavage daily for 4 weeks. Interestingly, compared with the vehicle group, fenofibrate treatment significantly rescued cardiac hypertrophy in WT and *Fabp3*-KO mice. More specifically, IVS and LVPW were markedly decreased following fenofibrate treatment (Figures 7C–E). Consistent with attenuated cardiac function, hypertrophic and fibrosis-related genes, such as *Anp, Bnp*, and *Col3a1*, were decreased after fenofibrate treatment, but not by the vehicle (Figure 7F). In addition, histochemical analysis showed that *Fabp3* deletion increased left ventricular wall thickness, cardiomyocyte size, and collagen volume, while fenofibrate supplement significantly rescued these effects whether in the WT or *Fabp3*-KO mice (Figures 7G–K).

**Figure 8:**
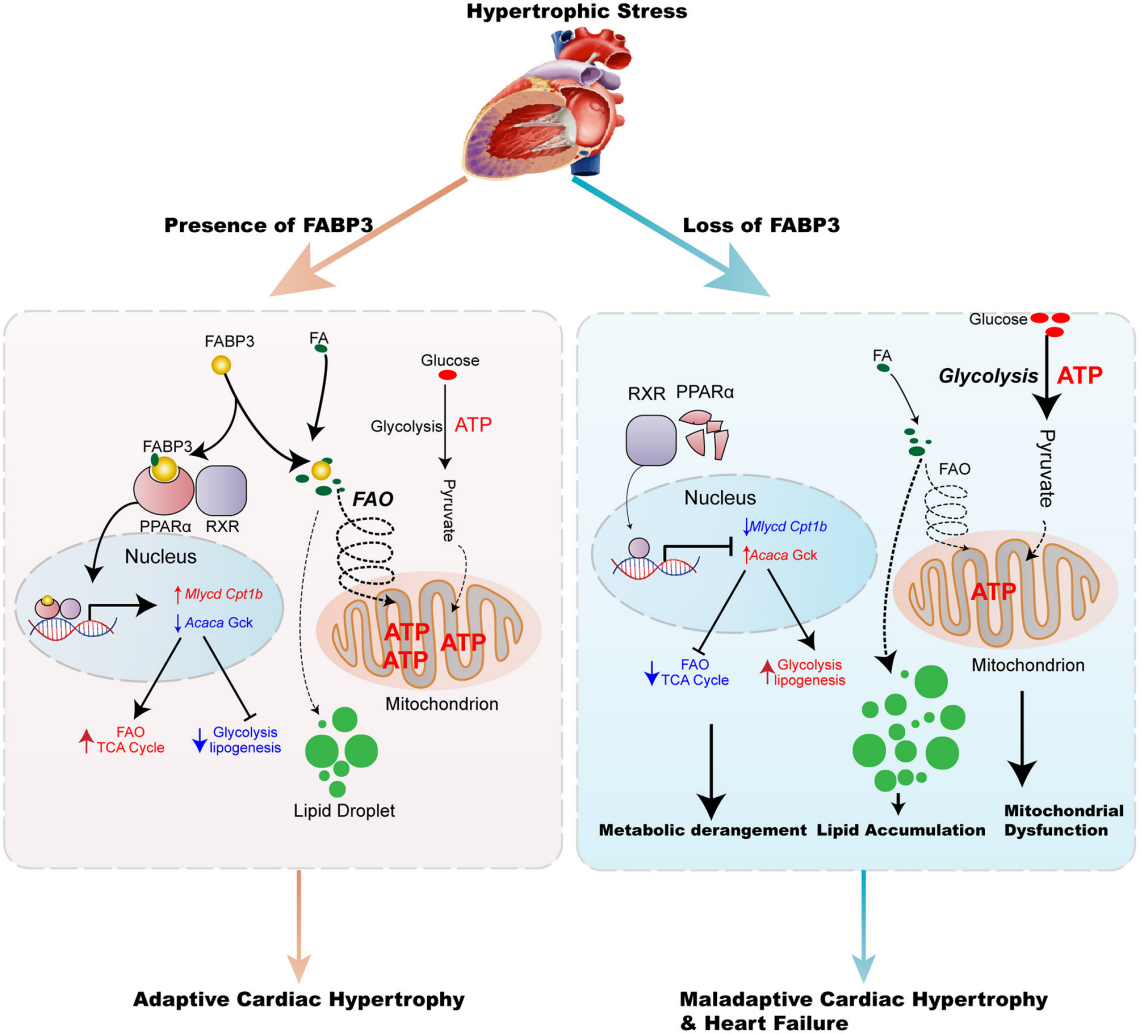
Model of the FABP3-mediated PPARα pathway in cardiac metabolic reprogramming and progression of cardiac hypertrophy and heart failure. The schematic demonstrates that the FABP3-mediated PPARα pathway regulates cardiac metabolic reprogramming by directly interacting with PPARα, thereby stabilizing and inducing its transactivation, which triggers downstream Mlycd and Cpt1b gene expression, while suppressing Acaca and Gck gene expression. This results in increased FAO levels and mitochondrial ATP production, ultimately resulting in adaptive cardiac function. However, in mice lacking FABP3, the response of FABP3-mediated PPARα on FAO/Glycolysis balance is abolished, and leads to the degradation of PPARα, lower transcriptional activity on FAO genes accompanied by increased lipogenesis, and glycolysis gene expression, which contributes to the increase in the glycolysis rate, accumulation of toxic lipid species, and compromised mitochondrial ATP production, thereby aggravating the progression of cardiac hypertrophy and heart failure.

Collectively, these results demonstrate an important role of the FABP3-PPARα pathway on fuel preference and cardiac hypertrophy, while treatment with fenofibrate may reverse hypertrophy, suggesting the potential clinical value for fenofibrate in hypertrophic treatment (Figure 8).

## Discussion

Herein, we observed an indispensable role of FABP3 in the incidence and advance of cardiac hypertrophy. *Fabp3* deficiency served to exacerbate TAC-induced hypertrophy and cardiac dysfunction, while its overexpression rescued cardiomyocyte hypertrophy. Mechanistically, *Fabp3* defect resulted in metabolic derangement characterized by defective FAO and increased glycolysis to elicit an imbalanced FAO/glycolysis rate and toxic lipid accumulation. Furthermore, *Fabp3* mediated the PPARα protein levels by directly binding to PPARα and preventing its degradation and underpinning its transactivation on *Mlycd* and *Gck*, which underscores the pivotal metabolic role of PPARα in FABP3-mediated cardiac hypertrophy. Additionally, treatment with the PPARα agonist, fenofibrate, effectively repressed *Fabp3*-KO induced cardiac hypertrophy, highlighting a potential clinical value of hypertrophic treatment by targeting cardiac energy metabolism.

FABP3, a small molecular weight protein, is abundantly expressed in the heart and muscle tissues (21). FABP3 can directly bind to cellular insoluble LCFAs and transport them to the mitochondrion, nucleus, or endoplasmic reticulum for utilization (12). Notably, single-cell RNA-sequencing research suggested that the increased expression of *Fabp3* from embryo to mature cardiomyocytes accounts for the developmental metabolic switch from embryonic glycolysis to postnatal mitochondrial fatty acid oxidation, underscoring its profound effects on fuel preference and cardiac metabolism (22). Besides that, the loss and gain function of FABP3 in brown adipocytes shows that FABP3 is a determinant of cellular fatty acid oxidation efficiency; brown adipocytes without FABP3 show defective capability to oxidize exogenously supplied fatty acids (16). These studies confirm the indispensable role of FABP3 on the maintenance of cardiac FAO and glucose homeostasis under physiological and pathological conditions.

In our previous studies, we reported that FABP3 plays an important role in myocardial infarction and in-stent restenosis (15, 23). Moreover, clinical studies have considered FABP3 to be a marker of cardiac ischemic injury, demonstrating that its expression is associated with major adverse cardiac outcomes and recurrent MI (24–28). As elevated FABP3 has been previously explored in the content of varied cardiac diseases, at least two mechanisms are involved in the regulation of FABP3 expression. Under stimulations, the myocardium shifts for higher energy demand, which occurs with the upregulation of IGF-1 and the decline of miR-1; (1) exposure to IGF-1 results in increased FABP3 expression, and (2) on the other hand, miR-1 downregulation is able to release its inhibitory role on FABP3 and eventually elicit the increase in FABP3 under cardiac hypertrophy (14). These literatures demonstrate a tight control of the FABP3 level by IGF-1 and miR-1. Consistently, we showed that FABP3 was increased in response to hypertrophic stimulations *in vivo* and *in vitro* to modulate cardiac metabolic homeostasis, especially the FAO and glucose oxidation processes under cardiac hypertrophy, a disease condition with higher energy demands. Here, we combined multi-omics analyses, such as RNA-seq and metabolomics, to reveal the indispensable role of FABP3 in governing the cardiac energy regulatory program by increasing FAO and inhibiting glycolysis under hypertrophic stimuli. Therefore, FABP3 has an important homeostatic role in the pathologically stressed mammalian heart. To the best of our knowledge, this is the first study to explore the role of FABP3 during cardiac hypertrophy.

As we have shown, *Fabp3* deletion contributes to dramatic abnormalities in myocardial metabolism by targeting PPARα pathways. Accumulating evidence has indicated that PPAR family members (PPARα, β/δ, and γ), a member of nuclear receptor superfamily of transcription factor (NR1C), play regulatory roles in cellular metabolism and cardiac hypertrophy, which was confirmed in loss- or gain-of-function mutated mice (29). Specifically, PPAR can directly bind to the promoter regions of metabolic-related genes and regulate their transcriptional levels (30). PPARγ is primarily expressed in adipose tissues, while PPARβ/δ is ubiquitously expressed. Of note, PPARα is exclusively expressed in tissues with higher capacity for fatty acid oxidation, such as the heart, liver, and BAT, which display a concordant expression profile with FABP3 (31). Cardiac-specific overexpression of PPARα resulted in the activation in FA transport genes and the suppression of glycolytic genes; however, these metabolic phenotypes were strikingly contrasted to the cardiac-specific overexpression of PPARβ/δ, which shows the increase in glycolysis and glucose uptake genes, indicating the reciprocal role of PPARα and PPARβ/δ on the regulation of cardiac metabolic homeostasis (32). Based on the expression profile and metabolic effects, the interactive effects of FABP3 and PPARα in cellular metabolism and cardiac hypertrophy were explored in this article. Previous studies show that the activation of PPARα exacerbates the uptake and utilization of FA and reduces glucose utilization (33, 34). Fasting or inhibiting mitochondrial FAO with etomoxir leads to markedly increased lipid accumulation in PPARα^−/−^ heart and hepatocytes (19). These phenotypes were similar to the derangement of the FAO/glucose oxidation rate in our FABP3-deficient mice, corroborating a link between FABP3 and PPARα. Moreover, studies have demonstrated a direct interaction between PPARα and FABP family members, especially FABP1 and FABP4, reporting that FABP1 or FABP4 translocates from the plasma to the nucleus, where it binds to PPARα to stabilize and amplify its biological function by enhancing its transcriptional activities (35–37). Additionally, a luciferase assay containing FABP3 and PPARα plasmid suggests that FABP3 facilitates the transcriptional activity of PPARα in COS-7 cells (38). However, whether FABP3 could interact with PPARα and govern its transcriptional activity in cardiomyocytes during chronic hypertrophic stimulation has not been fully delineated. Here, our *in vivo* and *in vitro* results reveal the potential mechanism of the FABP3-mediated PPARα pathway in cardiac hypertrophy and in FAO/glycolysis balance by directly binding to PPARα, promoting its stability, and underpinning its transactivation in *Mlycd* and *Gck*.

In conclusion, we have provided a novel perspective into the relationship between FABP3 with cardiac hypertrophy and fuel preference. However, in relation to our observations, there are some problems that need further explorations. We have demonstrated a beneficial value of FABP3 on cardiac hypertrophy and heart failure by inhibiting PPARα degradation. However, whether FABP3 mediated PPARα stability by preventing its degradation from ubiquitylation proteasome or other pathways needed further in-depth investigation. In conclusion, in the present study, we provide novel insights into the regulatory role of FABP3 on cellular metabolism following TAC-induced cardiac hypertrophy. Meanwhile, targeting FABP3 with an agonist may represent an attractive approach to alleviate deranged metabolic milieu in cardiac hypertrophy and achieve the goal of improved heart function.

## Data Availability Statement

All relevant data are included in the figures and Supplementary Materials. The scRNA-seq data presented in this article are available from the Gene Expression Omnibus (GEO) database under the accession number: GSE95143 (https://www.ncbi.nlm.nih.gov/geo/query/acc.cgi). Transcriptome data used in this study are deposited in the Gene Expression Omnibus (GEO) repository and are accessible through GEO series accession number: GSE177041.

## Ethics Statement

The animal study was reviewed and approved by Animal Care Committee of Shanghai Jiao Tong University School of Medicine.

## Author Contributions

XY, KC, and RT designed the study. LZ, ZL, and CL participated in the experimental design, data interpretation, and manuscript preparation. LZ, ZL, CL, QJ, LL, and KC discussed, edited, and revised the manuscript. ZL and YM participated in the revising and polishing of the re-submitted manuscript. All authors contributed to the article and approved the submitted version.

## Conflict of Interest

The authors declare that the research was conducted in the absence of any commercial or financial relationships that could be construed as a potential conflict of interest.

## Publisher's Note

All claims expressed in this article are solely those of the authors and do not necessarily represent those of their affiliated organizations, or those of the publisher, the editors and the reviewers. Any product that may be evaluated in this article, or claim that may be made by its manufacturer, is not guaranteed or endorsed by the publisher.
