# Supplementary material for: FABP3 Deficiency Exacerbates Metabolic Derangement in Cardiac Hypertrophy and Heart Failure via PPARα Pathway
Source: Front Cardiovasc Med. 2021 Aug 12;8:722908. doi: 10.3389/fcvm.2021.722908 (PMC8387950; doi:10.3389/fcvm.2021.722908)
Supplement: Supplementary file 1 [file Presentation_1.pdf]

Supplementary Material

Supplementary Materials & Methods

Supplementary Figures 1-8

Supplementary Tables 1-2

1. Supplementary Materials & Methods

1.1 Transverse aortic constriction (TAC) Model

The TAC model was used to induce pathological cardiac hypertrophy *in vivo* as described previously(1). Briefly, 8-week-old mice were anesthetized with isoflurane, intubated, and mechanically ventilated with a low concentration of isoflurane gas (1.0%). After Opening the sternum and moving aside the thymus glands, the aortic arch was visualized and ligated with 6-0 silk suture against a 27-gauge needle, then removed the needle and closed the chest with 5-0 silk suture. Mice were observed after 15 min for recovery. Sham-operated mice underwent an identical procedure except for aortic constriction.

1.2 Western blotting analyses

Total protein extracted from mice organs and cultured cells using SDS lysis (50 mM Tris, pH8.1, 1% SDS, sodium pyrophosphate, β-glycerophosphate, sodium orthovanadate, sodium fluoride, EDTA, and leupeptin) were used for western blot analysis as described previously(2). Antibodies against the following antigens were used: FABP3 (LS-C172162, LSBio, Seattle, WA, USA), GAPDH (97166, CST, Danvers, MA, USA), α-TUBULIN (66031-1-lg, Proteintech, Wuhan, Hubei, China), PPARα (sc-398394, Santa Cruz, Dallas, Texas, USA), ANP (sc-515701, Santa Cruz, Dallas, Texas, USA), MLYCD (15265-1-AP, Proteintech, Wuhan, Hubei, China), CPT1B (22170-1-AP, Proteintech, Wuhan, Hubei, China), ACC (67373-1-Ig, Proteintech, Wuhan, Hubei, China), GCK (19666-1-AP, Proteintech, Wuhan, Hubei, China). GAPDH and α-TUBULIN were used as controls. Densitometry values were measured by ImageJ for further analysis.

1.3 Real-time quantitative polymerase chain reaction (RT-qPCR)

Total RNA from mouse organs and cultured cells was extracted using Trizol reagent according to the manufacturer's protocols (Thermo Fisher Scientific Co., CA, USA). Primers for specific genes are presented in Supplementary Table. 2. The mRNA expression of indicated genes was amplified with ChamQ Universal SYBR qPCR Mix Kit (Q711-02, Vazyme, China) and detected using the Applied Biosystems QuantStudio 6 Flex Real-Time PCR System. The relative levels of specific genes were calculated by normalizing the level of *Gapdh* or *18S* using the $2^{-\Delta\Delta CT}$ method.

1.4 Immunocytochemistry and immunofluorescence staining (IF)

After antigen retrieval and permeabilization, cultured cells or heart sections were blocked with 5% BSA buffer for 30 min, following which the heart samples were probed overnight with primary antibodies. After washing with PBS and incubating with corresponding Alexa Fluor-conjugated secondary antibodies for 2 hours and DAPI for 5 min, the images were visualized using fluorescence microscopy and analyzed with ImageJ. Antibodies against the following antigens were used for IF staining: FABP3 (LS-C172162, LSBio, Seattle, WA, USA), PPARα (sc-398394, Santa Cruz, Dallas, Texas, USA), α-actinin (A7811, Sigma, St. Louis, MO, USA), and cTnT (GB11364, Servicebio, China). Quantification was performed using a minimum of three individual samples for each group, and a minimum of five high-resolution images were acquired for each

sample.

1.5 Echocardiography

Cardiac function was measured at 4 or 8 weeks after surgery using the Vevo 2100 High-Resolution Digital-Imaging System (Visual Sonics). After being anesthetized with 3% isoflurane, the mice were restrained on the warmed platform without isoflurane to obtain higher heart rates. The ventricular M and B-mode ultrasound at the papillary muscle level was measured to determine the heart size, thickness of the ventricular wall and septum, and evaluate cardiac function. All echo images captured and calculated were blinded to the researchers.

1.6 Histological analysis

Mice were anesthetized using isoflurane and transcardially perfused with PBS and 4% paraformaldehyde sequentially. Tissues were fixed overnight in paraformaldehyde and then embedded in paraffin or optimal cutting temperature compound (OCT) for histology and IF analysis. Standard hematoxylin and eosin (H&E) staining was performed for morphological comparison. The volume of ventricular collagen was assessed by Masson's trichrome staining and Sirius red staining using ImageJ. Notably, images of Sirius red staining were captured with fluorescence microscopy according to a previously described protocol(3). All experiments were performed at least in triplicate for achieving statistical significance.

1.7 Cardiomyocyte cross-sectional area analysis

Heart sections or cultured cells were stained with Wheat Germ Agglutinin (WGA, W11261, Invitrogen, Carlsbad, CA, USA) or α-actinin (A7811, Sigma, St. Louis, MO, USA) to determine the relative cell area *in vivo* and *in vitro,* respectively. The procedures of α-actinin staining were similar to IF assay as described previously. For WGA staining, heart sections were incubated with WGA working buffer (1:200) diluted in PBS for 20 min at room temperature after dewaxing and rehydration, following which the images were captured with fluorescence microscopy. To compare the myocyte cell area, images were acquired from at least three samples and 100 cells in hearts tissue and 50 cells in cultured dishes were measured per group using ImageJ.

1.8 Neonatal rat ventricular cardiomyocyte (NRVM) isolation

NRVMs were isolated from 1 to 3-day-old Sprague Dawley rats according to our lab protocols. In brief, hearts were cut into 1-3 mm pieces and digested with type II collagenase (0.2 mg/mL, Worthington Biochemical Corp, Freehold NJ, USA) diluted in HBSS buffer. After gently shaking at 37 °C for 7 min for 3–5 times, cardiomyocytes were released and plated with the cultured medium for 1.5 h to separate myocytes and fibroblasts. The myocytes were then re-plated with the culture medium (10% FBS, DMEM/F12, 1% penicillin/streptomycin) for 24 hours in a humidified atmosphere with 5% CO_2 at 37 °C.

1.9 Lentiviral constructs and transduction

cDNA including the full-length *Fabp3* gene (NM_001320996.1) was amplified via PCR, verified by sequencing, and cloned as a positive plasmid (LV-EF1a > FABP3/FLAG-CMV > eGFP/T2A/Puro). The negative control plasmid contained similar fragments as the positive control, while lacked the *Fabp3* element (LV-CMV > eGFP/T2A/Puro). H9C2 cells or NRVMs were transfected with lentivirus at an optimal multiplicity of infection of 10, and incubated with polybrene (5 μg/mL) for at least 24 hours, followed by selection with puromycin. The plasmid contained green fluorescent protein (GFP) allows for visualization under fluorescence microscopy.

1.10 Bulk RNA sequencing (RNA-seq)

WT and F3-KO hearts were collected at 1-week post-sham or TAC surgery and immediately frozen in liquid nitrogen. Total RNA was extracted from above tissues using Trizol according to standard protocols. Bioinformatics and significance analyses were performed in triplicate for statistical significance. Procedures of RNA-seq analysis, including RNA extraction, quantification, and cDNA library preparation, were performed by BGI Genomics procedures (BGI-SHENZHEN, China), using standard and consistent procedures.

1.11 RNA-seq data analysis

Sequencing data were filtered with SOAPnuke (v1.5.2)(4) to remove reads containing sequencing adapters and those with more than 1/5 low-quality base ratio. The resulting clean reads were saved in FASTQ format. The transcriptional reads were mapped to the reference genome using HISAT2 (v2.0.4)(5) and STAR (v2.3.0)(6). To identify differentially expressed genes, the expression of all mapped genes was calculated and analyzed based on their respective fragments per kilobase million (FPKM) value using the DESeq2 (v1.4.5)(7) with p value ≤ 0.05. Gene ontology (GO) (http://www.geneontology.org/) and KEGG (https://www.kegg.jp/) enrichment analyses of differentially expressed genes were performed using Phyper based on the hypergeometric test. The significant levels of terms and pathways were corrected by Q value with a rigorous threshold (Q value ≤ 0.05) by Bonferroni's test. Gene set enrichment analysis (GSEA) based on *c5: GO gene sets* dataset downloaded from the official website was performed(8). Genes related to fatty acid oxidation, glucose metabolism, TCA cycle, and lipid biogenesis were manually selected and plotted as the heatmap using all sample expression matrix in Rstudio with the package Pheatmap.

1.12 Transmission Electron Microscopy (TEM)

After perfusing with cold PBS, the left ventricles were dissected into 1-2 mm pieces, fixed immediately in 2.5% glutaraldehyde buffer at room temperature for 2 hours, and then stored overnight at 4 °C. A 70-90 nm thin section was cut and imaged using TEM. All TEM images were obtained by technicians that were blinded to the study design.

1.13 LC/GC-MS metabonomic analysis

WT and F3-KO mice were subjected to TAC surgery, after which their hearts were dissected at 1-week post-surgery and immediately frozen in liquid nitrogen. Approximately 50 mg of tissue was added to an extraction mix (methanol: water: chloroform = 6: 3: 4), vortexed for 30 s, homogenized

for 4 min, and ultra-sonicated for 5 min followed by centrifugation at 10,000 rpm for 15 min. After evaporation in a vacuum concentrator, all samples were analyzed using an Agilent 7890 GC-MS with a DB-5MS capillary column. Metabonomic analysis, including metabolite extraction and GC-MS analysis, annotation, and pathway enrichment analysis were performed by SHANGHAI BIOTREE Biomedical Technology Co., Ltd. according to standard procedures.

1.14 LC/GC-MS data annotation and analysis

The GC-MS raw data analysis, including peak extraction, baseline adjustment, deconvolution, alignment, and integration, was completed using the Chroma TOF (V 4.3x, LECO) software. The LECO-Fiehn Rtx5 database was used for metabolite identification by matching the mass spectrum and retention index. Finally, the peaks detected in less than half of the QC samples were removed(9). After data management and normalization, approximately 478 peaks were included. Firstly, principal component analysis (PCA) and orthogonal projections to latent structures-discriminant analysis (OPLS-DA)(10) was performed to compare the group variances in SIMCA software (V15.0.2, Sartorius Stedim Data Analytics AB, Umea, Sweden)(11). Next, differential metabolites were identified with p value < 0.05 using Student's *t*-test and variable importance in the projection (VIP) of OPLS-DA score > 1. In addition, commercial databases including KEGG pathway and MetaboAnalyst (http://www.metaboanalyst.ca/) were used for pathway enrichment analysis.

1.15 Plasma non-esterified fatty acid (NEFA) assay

To quantify the concentration of NEFA in mouse serum, the NEFA assay was performed according to the manufacturer's protocols (294-63601, Wako, Japan). Briefly, blood obtained from sham or TAC-operated mice was rested at room temperature for 2 hours followed by centrifugation (2000g, 20 min), after which the plasma was collected. Approximately 4 µL of serum was added to each well of the 96-well microplate and mixed with the reaction solution. Absorbance was then measured at 550 nm with a spectrophotometer.

1.16 Co-Immunoprecipitation (Co-IP)

NRVMs with a knocking-in expression of *Fabp3* or *Ppara* were treated with Ang II for 24 h. Cultured cells were then homogenized in IP/lysis buffer (50 mM Tris, 150 mM Nacl, 1% NP-40, 1 mM EDTA, PH 7.4) supplemented with PMSF and phosphatase inhibitor cocktails (Sigma-Aldrich). After centrifugation at 14,000*g* at 4 °C for 20 min, a total of 200 µg of whole cell lysate was incubated with 1 µg antibodies for 12 h at 4 °C. Next, the antibody, antigen, and magnetic bead complex were washed with IP/lysis buffer twice, boiled with 2x SDS-PAGE loading buffer (C508321, Sangon Biotech, Shanghai, China), and analyzed by western blot assay using corresponding antibodies. Normal mouse IgG was used as the negative control for immunoprecipitation.

1.17 Dual-luciferase activity assay

Pparα-driven promoter including three responsive elements (3x AGGACAAAGGTCA, $PPRE_3$-

TK-LUC), the *Mlycd* promoter (Mlycd-LUC) and the *Gck* promoter (Gck-LUC) was cloned into GV238 expression vectors. Renilla reporter plasmid was used as a negative control. HEK 293T cells were cultured in 48-well plates and transfected with 0.5 μg corresponding plasmid and 0.05 μg renilla vector for 6 h. After 24 h, luciferase activity in cultured cells was measured using the Dual-Luciferase@ Reporter assay system (E2920, Promega, Madison, WI) according to the manufacturer's instructions. Normalized firefly luciferase was obtained by dividing renilla luminescence units and was normalized to the ratio of the control group. Each experiment was performed with at least three samples for statistical significance.

1.18 Mitochondrial stress assay

Oxygen consumption rates were measured and calculated in the Seahorse XF24e analyzer. NRVMs were isolated and seeded into XF24e cell culture plates in a 2x 10^4 density and allowed to adhere overnight. Then cells were transfected with lentivirus containing FABP3 or its negative virus and kept for 3 days for selection and treatment with Ang II for 24 hours before OCR measurement. One hour before the recording, the culture medium was replaced as Seahorse XF DMEM medium with 10 mM glucose, 2 mM glutamine, 1 mM pyruvate. Then OCR was recorded as subsequential injection of the following compounds: Oligomycin, 1.5 mM; FCCP, 3 mM; Rot/AA, 0.5 mM (103015-100, XF Cell Mito Stress Test Kit, Agilent Technologies).

1.19 LCFA oxidation stress assay

NRVMs were isolated as described before and 4,000 cells / well were seeded into Seahorse XF96 cell culture plate and cultured overnight. Next day, cells were transfected with lentivirus containing FABP3 or its negative virus for 24 hours, then treated with Ang II for 24 hours before OCR measurement. One hour before the experiment, cell culture medium was changed to Seahorse XF DMEM medium with 10 mM glucose, 2 mM glutamine, 1 mM pyruvate at a final PH of 7.4 and placed in a non-CO2 incubator for 1 hour. The following concentrations for each pot were applied in our assays: Etomoxir, 4 mM; Oligomycin, 1.5 mM; FCCP, 3 mM; Rot/AA, 0.5 mM (103672-100, XF Cell Mito Stress Test Kit, Agilent Technologies).

1.20 Glycolytic rate analysis

Real-time measurement of extracellular acidification rate (ECAR) and oxygen consumption rate (OCR) enables to determine of the glycolytic proton efflux rate (glycoPER) in detected cells. A total of 4,000 NRVMs were seeded into Seahorse XF96 cell culture plate and cultured overnight. Next day, cells were transfected with either FABP3 siRNA or FABP3 lentivirus. One hour before the experiment, cell culture medium was changed to Seahorse XF DMEM medium with 10 mM glucose, 2 mM glutamine, 1 mM pyruvate at a final PH of 7.4 and placed in a non-CO2 incubator for 1 hour. The following concentrations for each pot were applied in our assays: Rot/AA, 0.5 mM; 2-DG, 50 mM (103344-100, XF Cell Mito Stress Test Kit, Agilent Technologies).

2.1 Supplemental Figures and Figure Legends

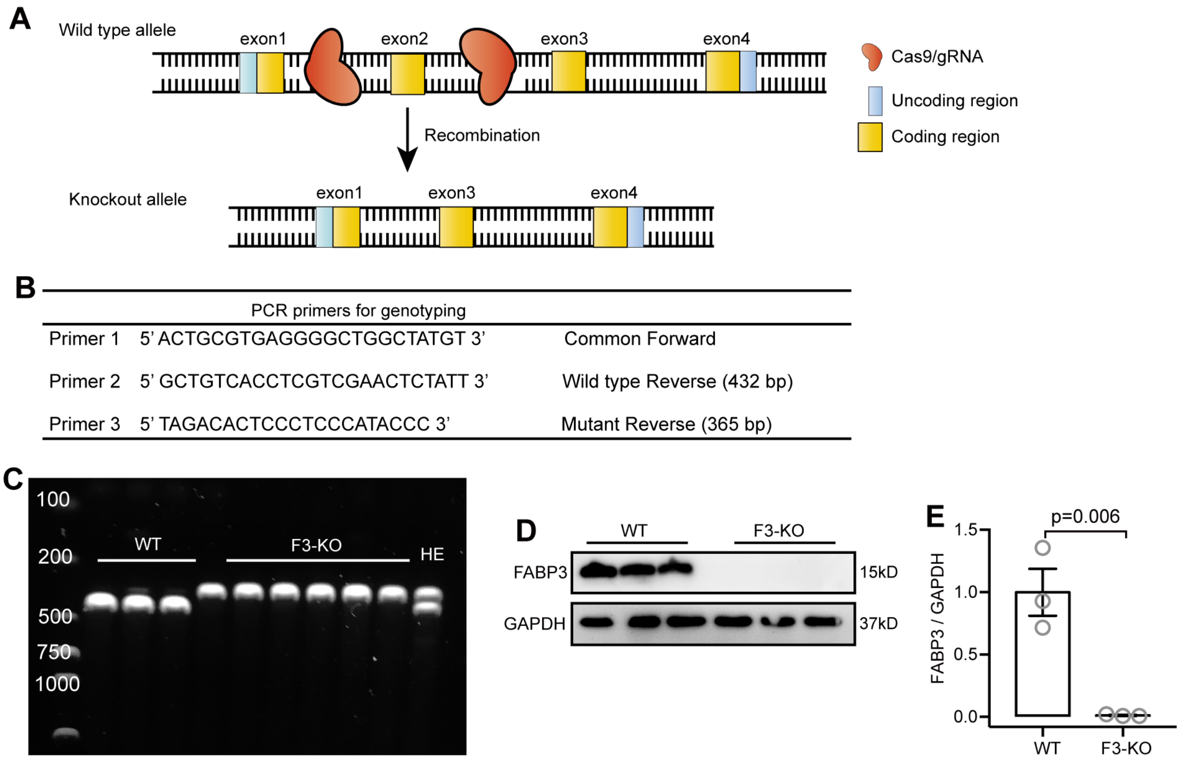

Supplementary Figure 1. Construction of *Fabp3*-knock-out mice with the CRISPR/Cas9 method. (A) Schematic diagram of *Fabp3* knock-out strategy. (B) Sequences of primers used for genotyping. Primers 1 and 2 amplify a 432-bp fragment in the wild-type (WT) allele, while primers 1 and 3 amplify a 365-bp fragment in the homozygous mutant allele. (C) Representative PCR genotyping images including WT, homozygous (F3-KO), and heterozygous mice (HE) mice. (D) Representative western blot images showing reduced FABP3 expression in *Fabp3*-null and its WT alleles. (E) Quantification of (D). [E, n = 3, Student's t-test.]

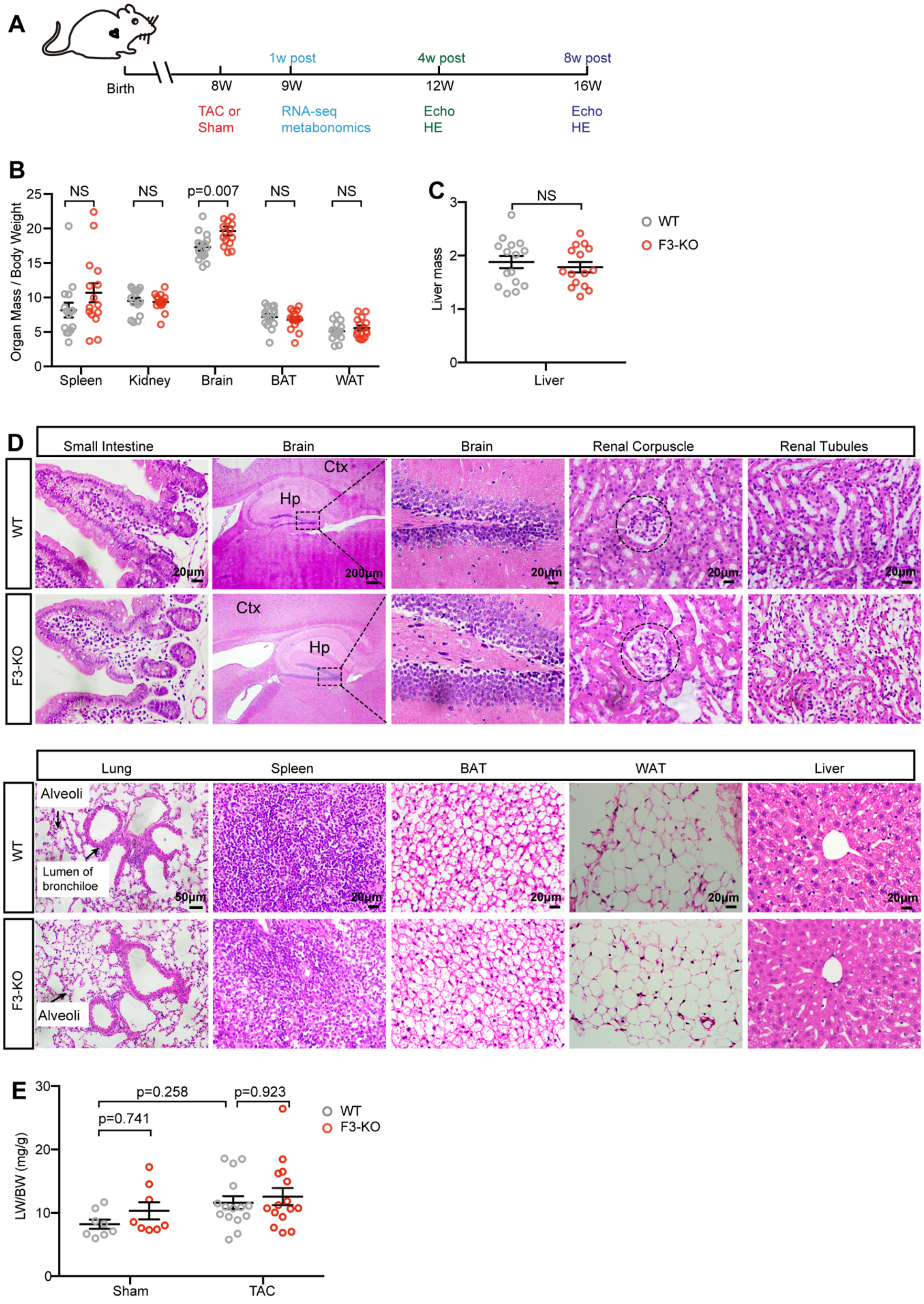
A
Birth
8W
9W
12W
16W
1w post
4w post
8w post
TAC or Sham
RNA-seq metabonomics
Echo HE
Echo HE
B
Organ Mass / Body Weight
NS
NS
p=0.007
NS
NS
Spleen
Kidney
Brain
BAT
WAT
C
Liver mass
NS
Liver
WT
F3-KO
D
Small Intestine
Brain
Brain
Renal Corpuscle
Renal Tubules
Ctx
Hp
WT
F3-KO
20μm
200μm
Lung
Spleen
BAT
WAT
Liver
Alveoli
Lumen of bronchiloe
50μm
E
LW/BW (mg/g)
p=0.258
p=0.923
p=0.741
Sham
TAC

Supplementary Figure 2. FABP3 defect does not affect tissue mass or their morphology except for hearts. (A) Experimental schematic diagram illustrating the strategy for multi-omics analysis, echocardiography, and morphology analysis of WT and F3-KO mice after TAC or sham surgery. (B) The ratio of organ mass to body weight in WT and F3-KO mice at 4 weeks after TAC operations. (C) Liver mass in F3-KO mice and its WT littermates. (D) Representative H&E images of tissue organs, including the small intestine, brain, kidney, lung, brown adipose tissue (BAT), and white adipose tissue (WAT), from WT and F3-KO mice at 4 weeks after TAC operations. (E) The ratio of lung weight to body weight from sham- or TAC-operated WT and F3-KO mice. [B, C, n = 15, Student's t-test; E, n = 8, 8, 15, 15, respectively, Tukey post-hoc test.]

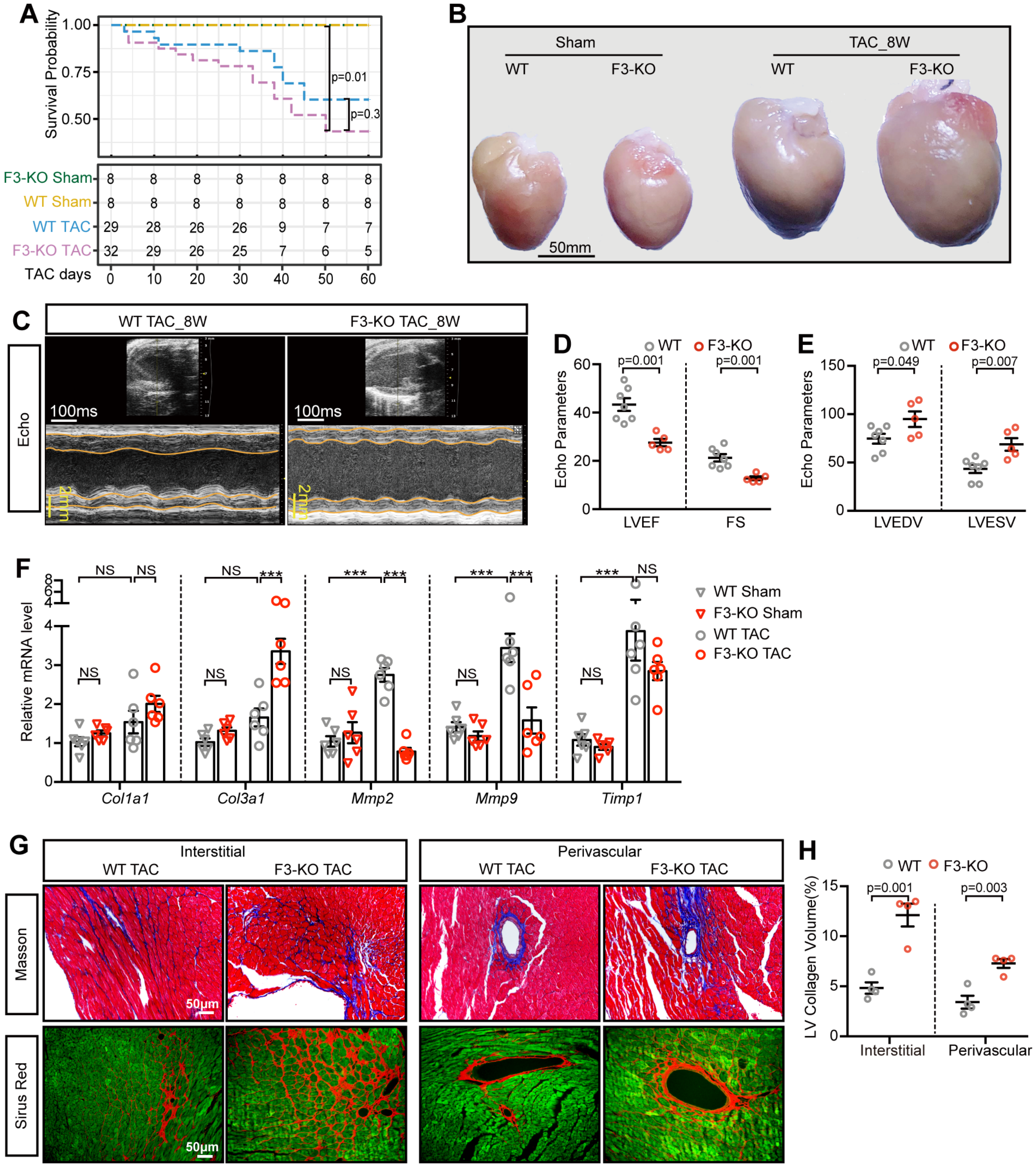

Supplementary Figure 3. FABP3-null exacerbates TAC-induced cardiac dysfunction and fibrosis. (A) Survival probability of WT and F3-KO mice up to 8 weeks after TAC or sham surgery. (B) Gross heart morphology images of WT and F3-KO mice at 8 weeks after surgery. (C) Representative echo images of WT and F3-KO mice at 8 weeks after TAC operation. (D) Quantification of left ventricular ejection fraction (LVEF) and fractional shortening (FS) in panel (C). (E) Quantification of left ventricular volume at diastole and systole (LVEDV, LVESV) in panel (C). (F) mRNA expression of fibrosis-related genes (*Col1a1*, *Col3a1*, *Mmp2*, *Mmp9*, and *Timp1*) from WT and F3-KO hearts at sham or 8 weeks post-surgery. NS, not significant, *** $p < 0.001$.

(G) Representative images of Masson (top) and Sirus Red staining (bottom) in interstitial and perivascular area of WT and F3-KO hearts. (H) Quantification results of collagen volume in panel (G). [D, E, n = 7, 5, respectively; F, n = 6, Tukey’s post-hoc test; H, n = 4; (D, E, and H): Student's t-test.]

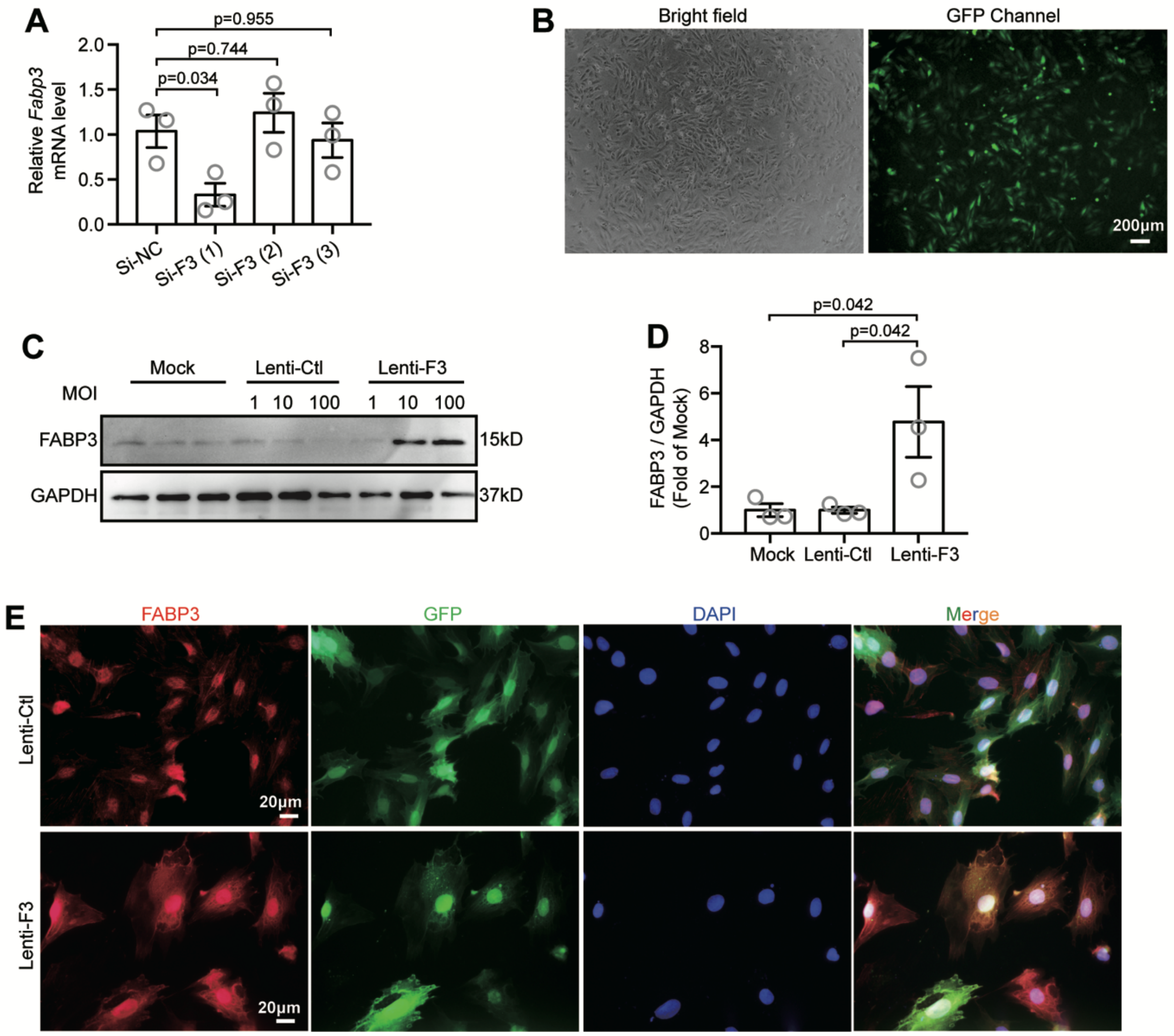

Supplementary Figure 4. Manipulating *Fabp3* expression with siRNA or lentivirus. (A) H9C2 cells were transfected with si-RNA targeting *Fabp3* (Si-F3(1), Si-F3(2), Si-F3(3)) or its negative control (Si-NC), then total RNA was extracted for qPCR analysis to determine *Fabp3* mRNA expression. (B) H9C2 cells were transfected with lentivirus and visualized using a microscope to determine the transfection efficiency; (Left) bright field of cultured cells, (Right) GFP channel of the same field corresponding to the left. (C) H9C2 cells were transfected with *Fabp3*-lentivirus or its control virus (Lenti-Ctl) at different MOIs (1, 10, 100). The whole cell lysates were then analyzed with western blotting. (D) Quantification results of (C). (E) Immunofluorescence co-staining of FABP3 (red), GFP (green), and DAPI (blue) after Lenti-Ctl or Lenti-F3 transfection. [A, D, n = 3; Dunnett's post-hoc test.]

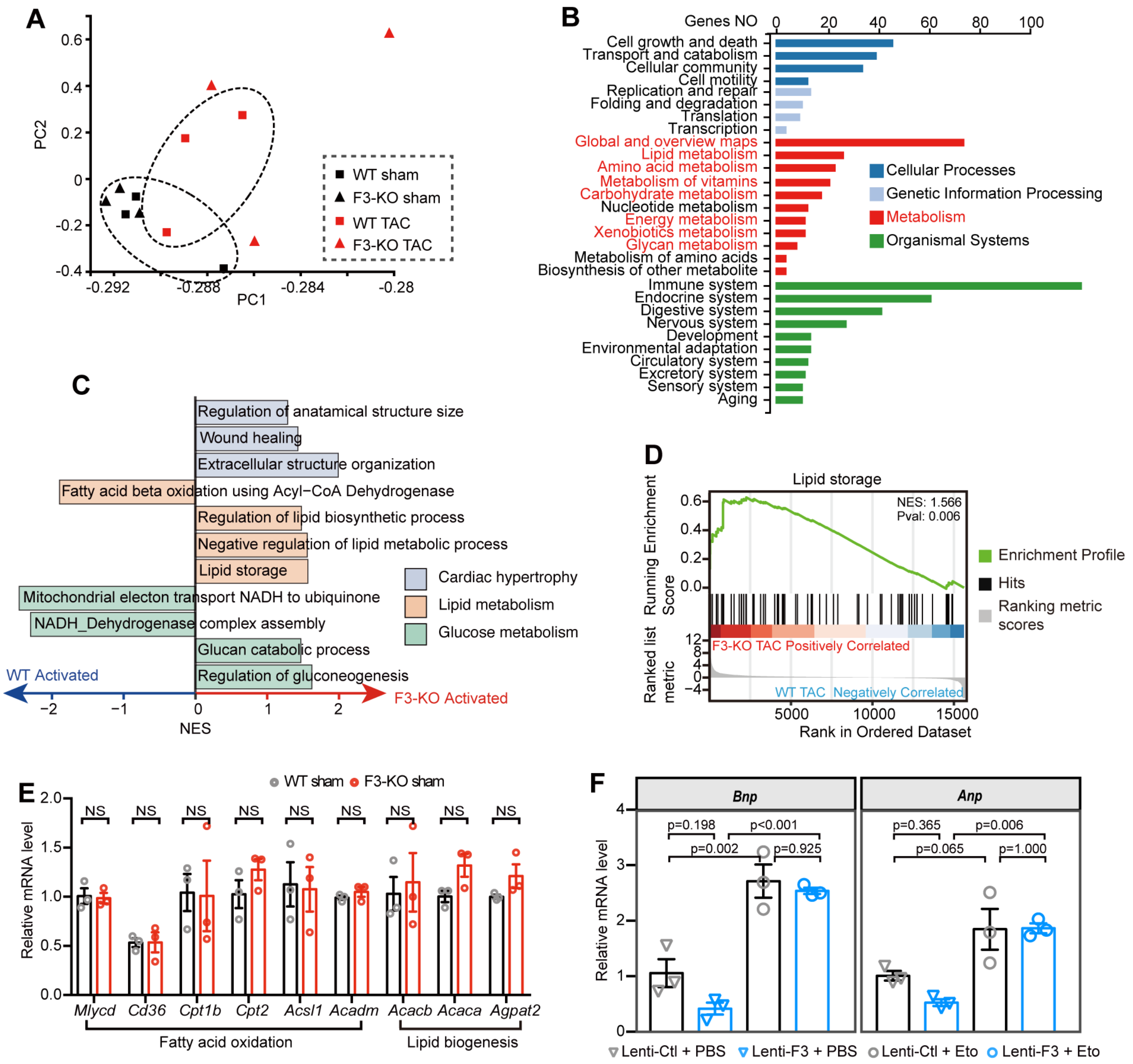

Supplementary Figure 5. FABP3-defect hearts exhibit deranged metabolic pathways. (A) PCA plot comprising all samples in RNA-seq analysis (n = 3 biological replicates / per group). (B) KEGG functional enrichment results including four biological terms (cellular process, genetic information processing, metabolism, and organismal systems) between TAC-operated F3-KO and WT hearts. (C) GSEA analysis based on GO terms comparing differentially activated pathways between TAC-operated F3-KO and WT hearts. Results are displayed according to the normalized enrichment score (NES). Pathways with positive NES indicated more upregulated genes in F3-KO hearts. (D) GSEA plot showed that *Fabp3*-null hearts are positively correlated with lipid storage. (E) The mRNA expression of FAO and lipid biogenesis genes in WT and F3-KO hearts after sham operations was determined by qPCR assay. NS, not significant. (F) NRVMs with overexpression of *Fabp3* were treated with or without etomoxir (Eto), then the mRNA expression of *Bnp* and *Anp* was determine by qPCR assay. [E, n = 3, Student's t-test; F, n = 3, Tukey’s post-hoc test.]

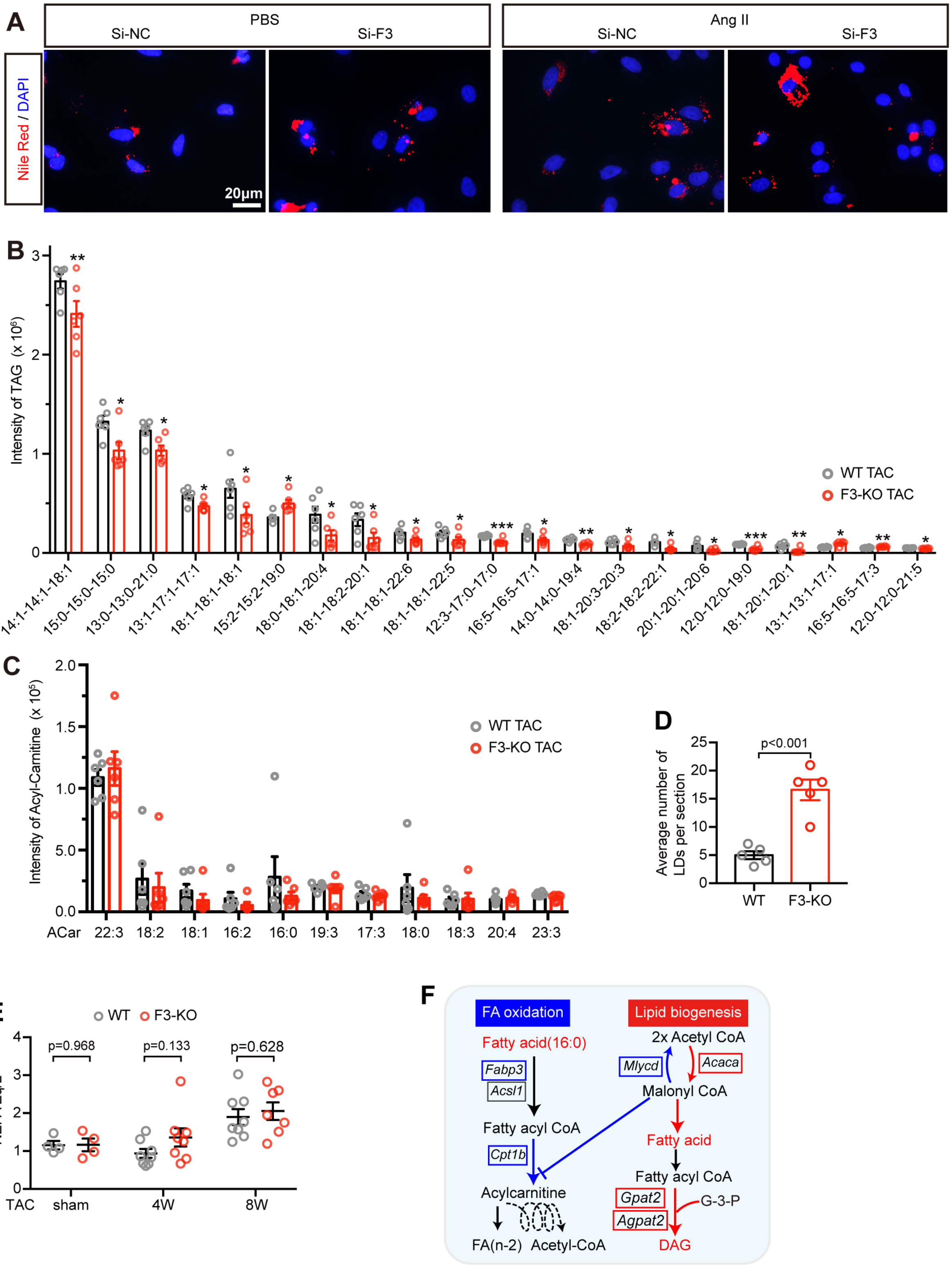

Supplementary Figure 6. FABP3-depletion leads to excessive cardiac lipid accumulation after TAC operation. (A) NRVMs with knocking-down expression of *Fabp3* or its scrambled control were stained with nile red and DAPI (blue). (B) The level of triglyceride (TAG) in WT and

F3-KO hearts was determined by LC-MS analysis, 6 biological replicates / per group. * $p < 0.05$, ** $p < 0.01$. (C) The level of Acyl-Carnitine (ACar) in TAC-operated WT and F3-KO hearts was determined by LC-MS analysis. (D) Quantifying the number of lipid droplet in Figure 4L. (E) Blood non-esterified fatty acid (NEFA) levels were measured in F3-KO and WT littermates at 0, 4, and 8 weeks after TAC operations. (F) Schematic diagram including FAO/lipid biogenesis genes and metabolites shows defective FAO and activated lipid biogenesis in F3-KO hearts. Red font and rectangles denote upregulated metabolites or genes, and blue represents those that are downregulated. [D, n = 5; E, n = 4, 4, 8, 8, 8, 7, respectively; (B, D and E): Student's t-test.]

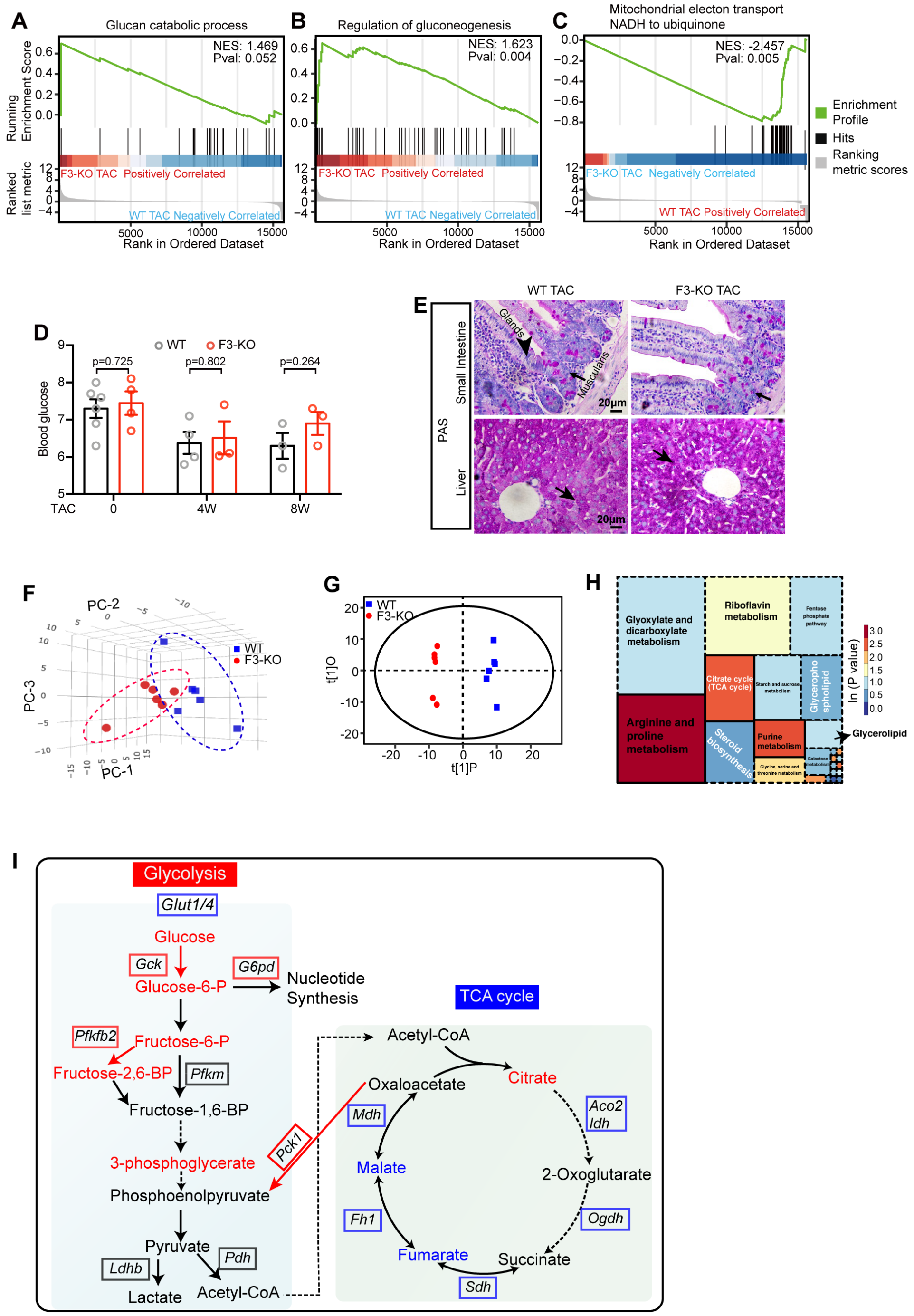

Supplementary Figure 7. FABP3-null hearts show increased glycolysis after TAC surgery. (A-C) GSEA plot showed *Fabp3*-defect heart was positively correlated with “glucan catabolic process” (A), “Regulation of gluconeogenesis” (B), while was negatively correlated with “Mitochondrial electon transport NADH to ubiquinone” (C). (D) Blood glucose level was measured in F3-KO mice and WT littermates at 0-week, 4-weeks, and 8-weeks after TAC operation.

(E) Periodic acid Schiff glycogen staining (PAS) of the small intestine and liver sections was performed on TAC-operated WT and F3-KO mice at 4 weeks post-surgery. (F-H) Non-targeted metabolomics analysis was performed on TAC-operated WT and F3-KO hearts. (F) PCA plot showing metabolic differences in the above groups, n = 6 biological replicates/group. (G) Orthogonal partial least- discrimination analysis (OPLS-DA) based on differential metabolomics. (H) KEGG pathway analysis of differential metabolites. (I) Schematic diagram including glycolysis/TCA cycle genes and metabolites. Red font and rectangles denote upregulated metabolites or genes, and blue represents those that are downregulated. [D, n = 6, 4, 4, 3, 3, 3, respectively, Student's t-test.]

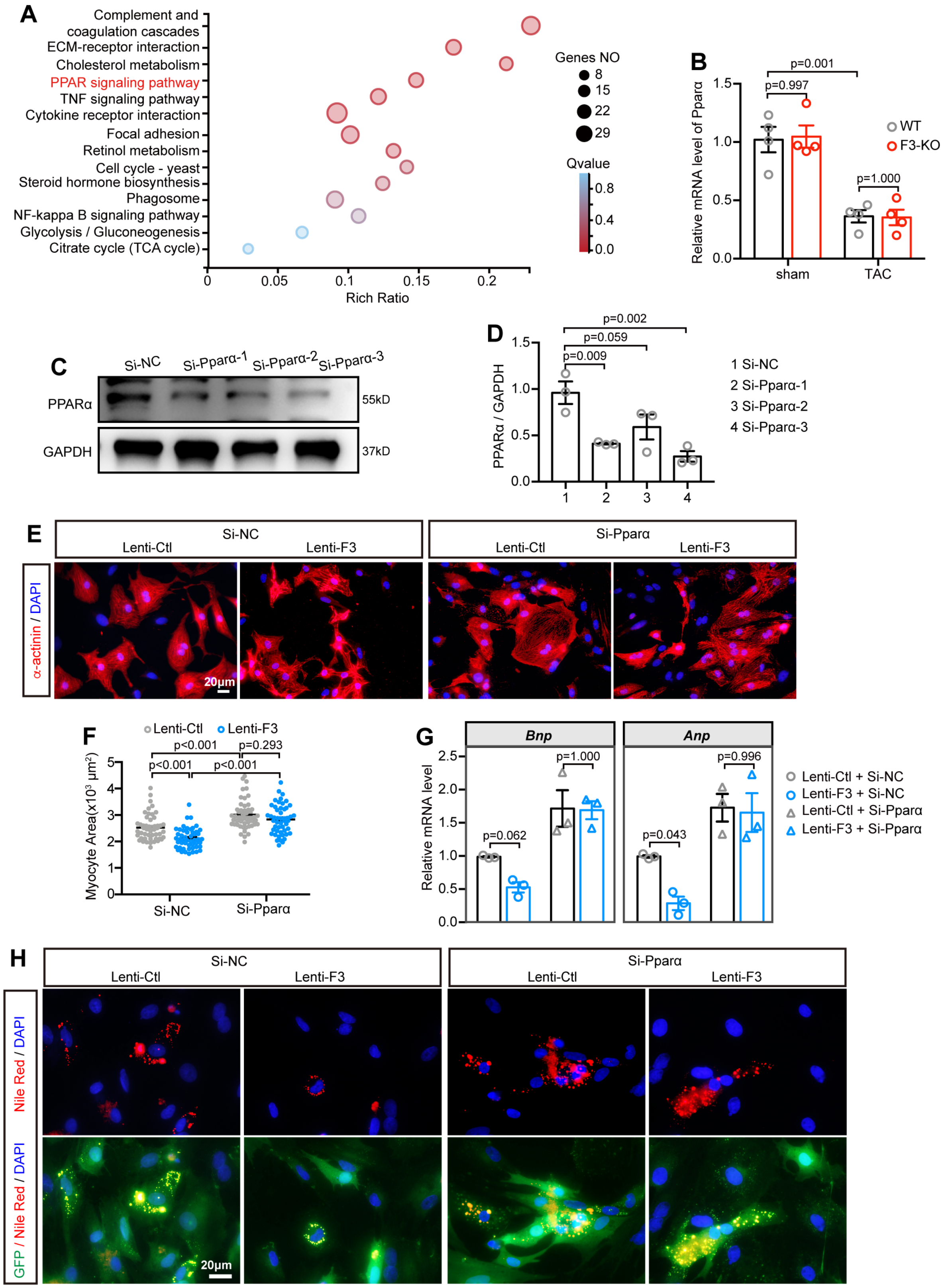

A
Complement and coagulation cascades
ECM-receptor interaction
Cholesterol metabolism
PPAR signaling pathway
TNF signaling pathway
Cytokine receptor interaction
Focal adhesion
Retinol metabolism
Cell cycle - yeast
Steroid hormone biosynthesis
Phagosome
NF-kappa B signaling pathway
Glycolysis / Gluconeogenesis
Citrate cycle (TCA cycle)
Rich Ratio
Genes NO
Qvalue
B
Relative mRNA level of Pparα
p=0.001
p=0.997
p=1.000
WT
F3-KO
sham
TAC
C
Si-NC
Si-Pparα-1
Si-Pparα-2
Si-Pparα-3
PPARα
GAPDH
55kD
37kD
D
PPARα / GAPDH
p=0.002
p=0.059
p=0.009
1 Si-NC
2 Si-Pparα-1
3 Si-Pparα-2
4 Si-Pparα-3
E
Si-NC
Si-Pparα
Lenti-Ctl
Lenti-F3
α-actinin / DAPI
20μm
F
Lenti-Ctl
Lenti-F3
Myocyte Area(x10³ μm²)
p<0.001
p=0.293
p<0.001
p<0.001
Si-NC
Si-Pparα
G
Bnp
Anp
Relative mRNA level
p=1.000
p=0.062
p=0.996
p=0.043
Lenti-Ctl + Si-NC
Lenti-F3 + Si-NC
Lenti-Ctl + Si-Pparα
Lenti-F3 + Si-Pparα
H
Nile Red / DAPI
GFP / Nile Red / DAPI
20μm

Supplementary Figure 8. Requirement of PPARα in FABP3-driven metabolic regulation and cardiac hypertrophic response. (A) KEGG analyses of top enriched pathways based on differentially expressed genes between TAC-operated WT and F3-KO hearts in RNA-seq analysis. (B) The mRNA expression of *Pparα* in WT and F3-KO hearts after sham- or TAC-operation. (C) NRVMs were transfected with siRNA targeting *Pparα* or its negative control and its protein expression was determined by western blot assay. (D) The quantification results in panel (C). (E) NRVMs with knocking-in expression of FABP3 were transfected with Si-Pparα or its negative control. After Ang II treatment, cell area was determined by a-actinin staining. (F) The quantification results of cell area in (E), 50 cells /group were calculated. (G) The mRNA expression of *Bnp* and *Anp* was determined in the indicated groups by qPCR assay. (H) The level of neutral lipid was measured by nile red staining in the aforementioned groups. [D, n = 3, Dunnett's post-hoc test; F, Tukey's post-hoc test; G, n = 3, Games-Howell post-hoc test.]

2.2 Supplementary Tables

Supplementary Table 1. Expression of PPARα target genes in RNA-seq

Supplementary Table 2. Primer pairs used in the current study

Supplementary Table 1. Expression of PPARα target genes in RNA-seq

MUS ID	Terms	log2(F3-KO TAC vs WT TAC)	Qvalue(F3-KO TAC v WT TAC)	log2(F3-KO sham vs WT sham)	Qvalue(F3-KO sham vs WT sham)
Gck	glycolysis	1.694E+00	2.000E-181	1.201E+00	4.060E-54
Pklr	glycolysis	3.834E+00	1.774E-02	NA	NA
Pck1	glycolysis	3.696E+00	2.420E-08	-1.656E+00	4.542E-01
G6pc	glycolysis	3.419E+00	5.425E-02	NA	NA
Fbp1	glycolysis	2.319E+00	3.020E-04	-4.861E-01	7.884E-01
Pcx	glycolysis	-1.220E-01	6.733E-02	-1.657E-02	8.640E-01
G6pc3	glycolysis	-9.954E-02	6.387E-02	-9.750E-02	9.396E-02
Fbp2	glycolysis	-1.531E+00	1.800E-84	-6.448E-01	9.570E-20
Pdk4	glycolysis	-1.856E+00	0.000E+00	-4.201E-01	2.250E-52
Eno1	glycolysis	-1.871E-01	1.010E-14	1.613E-01	3.140E-09
Cpt1b	Mitochondrial β-Oxidation	-2.654E-01	6.160E-100	1.183E-01	9.380E-21
Mlycd	Mitochondrial β-Oxidation	-4.021E-01	1.030E-31	1.103E-02	8.401E-01
Acaa2	Mitochondrial β-Oxidation	-5.587E-01	0.000E+00	2.349E-01	7.740E-85
Acadl	Mitochondrial β-Oxidation	-2.831E-01	4.260E-165	-1.081E-01	4.980E-23
Acadm	Mitochondrial β-Oxidation	-2.022E-01	1.130E-82	6.708E-02	7.260E-11
Acads	Mitochondrial β-Oxidation	-1.543E-01	1.000E-07	9.716E-02	6.040E-04
Acadvl	Mitochondrial β-Oxidation	-3.518E-01	2.400E-211	2.211E-01	1.990E-98
Cpt2	Mitochondrial β-Oxidation	-2.880E-01	3.630E-33	5.199E-02	4.732E-02
Crat	Mitochondrial β-Oxidation	-2.193E-01	1.030E-53	5.248E-02	2.990E-04
Ehhadh	Mitochondrial β-Oxidation	-2.113E-01	1.672E-02	1.330E-01	2.390E-01

Hadha	Mitochondrial β-Oxidation	-3.991E-01	0.000E+00	1.681E-01	3.690E-102
Hadhb	Mitochondrial β-Oxidation	-1.304E-01	3.480E-54	2.792E-01	4.190E-295
Slc25a20	Mitochondrial β-Oxidation	-3.290E-01	7.040E-37	4.489E-01	2.170E-76
Aldh9a1	ω-Hydroxylation/Oxidation	-3.810E-01	6.690E-14	4.407E-02	5.159E-01
Cpt1a	Mitochondrial β-Oxidation	2.983E-01	2.310E-14	4.334E-01	2.800E-24
Acaca	Lipogenesis	3.354E-01	5.150E-05	-6.874E-02	5.780E-01
Agpat2	Lipogenesis	1.148E-01	2.359E-02	2.451E-01	1.540E-06
Elovl6	Lipogenesis	3.091E-01	3.578E-01	6.277E-01	1.130E-01
Fads1	Lipogenesis	2.607E-01	1.100E-05	-6.364E-02	5.038E-01
Fads2	Lipogenesis	5.966E-01	7.180E-08	8.289E-02	6.642E-01
Fasn	Lipogenesis	3.305E-01	1.640E-06	-6.352E-02	5.088E-01
Gpam	Lipogenesis	3.065E-01	5.230E-39	1.468E-01	5.160E-09
Scd1	Lipogenesis	3.667E-01	1.920E-06	-3.301E-01	1.905E-03
Scd2	Lipogenesis	3.173E-01	7.250E-06	-8.918E-02	3.801E-01
Acacb	Lipogenesis	-1.605E-01	6.250E-19	8.186E-02	6.280E-06
Apoa1	Lipid Binding & Transport / Lipoproteins	5.076E+00	3.440E-50	NA	NA
Apoa2	Lipid Binding & Transport / Lipoproteins	3.238E+00	4.910E-39	7.292E-01	1.633E-01
Apoa5	Lipid Binding & Transport / Lipoproteins	5.589E+00	7.080E-07	NA	NA
Apoc3	Lipid Binding & Transport / Lipoproteins	5.921E+00	2.080E-08	NA	NA
Lipc	Lipid Binding & Transport / Lipoproteins	5.709E-01	5.528E-01	-7.110E-02	9.869E-01

Lpl	Lipid Binding & Transport / Lipoproteins	-9.695E-02	2.320E-132	-2.253E-01	0.000E+00
Pltp	Lipid Binding & Transport / Lipoproteins	3.134E-01	1.670E-11	-6.226E-02	3.442E-01
Slc27a1	Lipid Binding & Transport / Lipoproteins	-9.675E-01	4.640E-264	-4.333E-01	8.650E-72
Slc27a2	Lipid Binding & Transport / Lipoproteins	2.082E+00	1.330E-07	-6.878E-01	4.459E-01
Slc27a4	Lipid Binding & Transport / Lipoproteins	-1.165E-02	9.063E-01	-1.734E-01	2.560E-02
Vldlr	Lipid Binding & Transport / Lipoproteins	1.692E-01	1.920E-23	6.631E-02	4.440E-04
Hmgcs2	Ketogenesis	-6.357E-01	3.470E-24	-6.318E-01	3.350E-16
Abca1	Cholesterol Metabolism	6.041E-01	3.630E-40	-6.307E-02	3.825E-01
Abca4	Cholesterol Metabolism	-2.034E+00	3.870E-29	2.817E-01	9.389E-02
Cyp7a1	Cholesterol Metabolism	2.834E+00	3.998E-02	-2.067E+00	2.870E-01
Cyp8b1	Cholesterol Metabolism	1.156E+00	1.737E-02	-6.561E-01	5.556E-01
Nr1h2	Cholesterol Metabolism	-9.100E-02	6.145E-02	-8.460E-02	1.108E-01
Nr1h3	Cholesterol Metabolism	-8.302E-02	4.458E-01	-6.667E-01	1.460E-14
Fgf12	growth factor activity	1.303E+00	7.757E-02	3.888E-01	6.388E-01
Acaa1a	Peroxisomal β-Oxidation	-1.459E-01	4.556E-03	-9.915E-03	9.047E-01
Acaa1b	Peroxisomal β-Oxidation	4.564E+00	2.370E-11	NA	NA
Acox1	Peroxisomal β-Oxidation	-7.232E-02	3.100E-05	-8.613E-02	5.550E-07
Ech1	Peroxisomal β-Oxidation	-5.972E-01	0.000E+00	-7.171E-02	5.500E-14

Supplementary Table 2. Primer pairs used in the current study

Primers for mouse qPCR		
Gene	Forward primers (5'- 3')	Reverse primers (5'- 3')
Fabp3	AGCCTGGACCCAGTTCCTAC	GGTGGCCTTGGTTCTGCTTTAT
Anp	GGGTAGGATTGACAGGATTGG	CTCCTTGGCTGTTATCTTCGG
Bnp	TGGGAGGTCACTCCTATCCT	GGCCATTTCCTCCGACTTT
Acta1	CCAAAGCTAACCGGGAGAAG	GACAGCACCGCCTGGATAG
Myh7	CGGACCTTGGAAGACCAGAT	GACAGCTCCCCATTCTCTGT
Col1a1	CATAAAGGGTCATCGTGGCT	TTGAGTCCGTCTTTGCCAG
Col3a1	ACGTAGATGAATTGGGATGCAG	GGGTTGGGGCAGTCTAGTG
Mmp2	ACCAAGAACTTCCGATTATCCC	CAGTACCAGTGTCAGTATCAGC
Mmp9	TCCCCAAAGACCTGAAAACC	CTGCTTCTCTCCCATCATCTG
Timp1	CTCAAAGACCTATAGTGCTGGC	CAAAGTGACGGCTCTGGTAG
Gapdh	GCCTTCCGTGTTCCTACC	CCTCAGTGTAGCCCAAGATG
Mlycd	CGCCTATCCCTGGATTCACC	ATCCCTGAGGTGCCAAACAC
Cd36	ATTCCCTTGGCAACCAACCA	TACGTGGCCCGGTTCTACTA
Cpt1b	GCACACCAGGCAGTAGCTTT	CAGGAGTTGATTCCAGACAGGTA
Cpt2	TGATGGCTGAGTGCTCCAAA	GAACACCAATGTTCATGAGGAAGAA
Acsl1	CGGCCGCGACTCCTTAAATA	ATAGGGCTGGTTTGGCTTCC
Acadm	GCGGCCATTAAGACCAAAGC	GAAGACAGGTTCCTCCGCCA
Acaca	ATGCACAGGACTGAGAAGGC	GTGATAAGGTGGTGGCAGGG
Acacb	TCCAAGTGGCCCTAGTGAGT	CGGATCCAGAGTGTTCGAGG

Agpat2	CAGCCAGGTTCTACGCCAAG	TGATGCTCATGTTATCCACGGT
Gck	TAGCGGGGGTCATAAATCGC	GCAGCCCTTACTCTTCTGGG
Pck1	TTGAACTGACAGACTCGCCC	GGCACTTGATGAACTCCCCA
Slc2a1	GCTTGTAGAGTGACGATCTGAGC	AAGCCAAACACCTGGGCAAT
Slc2a4	GCTCTGACGATGGGGAACC	CACCGAGACCAACGTGAAGA
Pparα	ACACGCGTGCGAGTTTTCA	TCGCCGAAAGAAGCCCTTAC
18S	GTAACCCGTTGAACCCCATT	CCATCCAATCGGTAGTAGCG

**Primers for Rat qPCR**		
Gene	Forward primers (5’- 3’)	Reverse primers (5’- 3’)
Fabp3	ATGAAGTCACTCGGTGTGGG	TCCCACTTCTGCACATGGAC
Anp	GAAGATGCCGGTAGAAGATGAG	AGAGCCCTCAGTTTGCTTTTC
Bnp	GGTGCTGCCCCAGATGATT	CTGGAGACTGGCTAGGACTTC
Myh7	GCCCCAAATGCAGCCAT	CGCTCAGTCATGGCGGAT
Gapdh	TGACAACTCCCTCAAGATTGTCA	GGCATGGACTGTGGTCATGA

Supplementary References:

[1] M. Zhang, H. Gao, D. Liu, X. Zhong, X. Shi, P. Yu, et al. (2019). CaMKII-delta9 promotes cardiomyopathy through disrupting UBE2T-dependent DNA repair. Nat Cell Biol. 21: 1152-1163. doi: 10.1038/s41556-019-0380-8.

[2] L.F. Zhuang, C.N. Li, Q.J. Chen, Q. Jin, L.Q. Wu, L. Lu, et al. (2019). Fatty acid-binding protein 3 contributes to ischemic heart injury by regulating cardiac myocyte apoptosis and MAPK pathways. Am J Physiol-Heart C. 316: H971-H984. doi: 10.1152/ajpheart.00360.2018.

[3] B. Vogel, H. Siebert, U. Hofmann, and S. Frantz. (2015). Determination of collagen content within picrosirius red stained paraffin-embedded tissue sections using fluorescence microscopy. MethodsX. 2: 124-34. doi: 10.1016/j.mex.2015.02.007.

[4] R. Li, Y. Li, K. Kristiansen, and J. Wang. (2008). SOAP: short oligonucleotide alignment program. Bioinformatics. 24: 713-714. doi,

[5] D. Kim, B. Langmead, and S.L. Salzberg. (2015). HISAT: a fast spliced aligner with low memory requirements. Nat Method. 12: 357-360. doi: HISAT: a fast spliced aligner with low memory requirements.

[6] A. Dobin, C.A. Davis, F. Schlesinger, J. Drenkow, C. Zaleski, S. Jha, et al. (2013). STAR: ultrafast universal RNA-seq aligner. Bioinformatics. 29: 15-21. doi: 10.1093/bioinformatics/bts635.

[7] M.I. Love, W. Huber, and S. Anders. (2014). Moderated estimation of fold change and dispersion for RNA-seq data with DESeq2. Genome Biol. 15: 550. doi: 10.1186/s13059-014-0550-8.

[8] A. Subramanian, P. Tamayo, V.K. Mootha, S. Mukherjee, B.L. Ebert, M.A. Gillette, et al. (2005). Gene set enrichment analysis: a knowledge-based approach for interpreting genome-wide expression profiles. Proc Natl Acad Sci U S A. 102: 15545-15550. doi: 10.1073/pnas.0506580102.

[9] W.B. Dunn, D. Broadhurst, P. Begley, E. Zelena, S. Francis-McIntyre, N. Anderson, et al. (2011). Procedures for large-scale metabolic profiling of serum and plasma using gas chromatography and liquid chromatography coupled to mass spectrometry. Nat Protoc. 6: 1060. doi: 10.1038/nprot.2011.335.

[10] J. Trygg, and S. Wold. (2002). Orthogonal projections to latent structures (O-PLS). J Chromatogr A. 16: 119-128.

[11] H. Tsugawa, Y. Tsujimoto, M. Arita, T. Bamba, and E. Fukusaki. (2011). GC/MS based metabolomics: development of a data mining system for metabolite identification by using soft independent modeling of class analogy (SIMCA). BMC bioinformatics. 12: 131. doi: 10.1186/1471-2105-12-131.
